# Knockouts of Sulfur Metabolism Genes Induce Chronic Inflammation and Immune Dysregulation in *Drosophila melanogaster*

**DOI:** 10.3390/antiox15070881

**Published:** 2026-07-16

**Authors:** Victoria Shilova, Alexander Rezvykh, Victoria Aristova, Artem Davletshin, Anna Andreeva, Ksenia Ponomareva, David Garbuz, Michael Evgen’ev, Olga Zatsepina

**Affiliations:** 1 Engelhardt Institute of Molecular Biology, Russian Academy of Sciences, 119991 Moscow, Russia; vika-shilova@yandex.ru (V.S.); aprezvykh@yandex.ru (A.R.); aristovaavictoriaa@gmail.com (V.A.); artem.dav7@yandex.ru (A.D.); wooflit@gmail.com (A.A.); dgarbuz@yandex.ru (D.G.); misha672011@yahoo.com (M.E.); 2 Moscow Center for Advanced Studies, 123592 Moscow, Russia; 3 Center for Master’s Programs, Master’s Program “Synthetic Biology and Biodesign”, First Moscow State Medical University, 119991 Moscow, Russia; newbox0003@gmail.com

**Keywords:** *Drosophila*, sulfur metabolism, inflammation, immune pathways

## Abstract

Hydrogen sulfide (H_2_S) is a vital gasotransmitter essential for maintaining redox homeostasis and modulating inflammatory responses. Herein, we examined a collection of *Drosophila melanogaster* knockout (KO) lines generated in our laboratory, lacking key genes involved in transsulfuration (*cbs*, *cse*) and sulfide metabolism (*tst1*), to elucidate the role of this adaptive system in immunity. Genetic ablation of these pathways results in profound H_2_S deficiency and hyperhomocysteinemia, leading to chronic oxidative stress. This leads to constitutive activation of major immune pathways—including IMD, Toll, and JAK-STAT—even in the absence of infection, a hallmark of chronic inflammation. Indeed, transcriptomic analysis and qRT-PCR studies revealed significant upregulation of these pathways in double (*cbs*; *cse*) and triple (*cbs*; *cse*; *tst1*) KO flies under control conditions. Following septic injury with *Bacillus subtilis*, double and triple KO flies exhibited increased expression of antimicrobial peptides (AMPs) and pattern recognition receptors compared to control and *TST1-/-* single KO lines. Notably, double and triple KO lines showed more prolonged immune activation. The triple KO flies exhibited the worst survival rate following septic injury. These results demonstrate that H_2_S deficiency causes chronic inflammation through the sustained activation of immune pathways, highlighting sulfur metabolism as a crucial regulator of homeostasis and innate immunity in *Drosophila*.

## 1. Introduction

Innate immunity represents the first and most evolutionarily conserved line of defense against invading pathogens [1]. In multicellular organisms, this system provides reliable and rapid protection against various infections, while avoiding significant collateral damage from chronic or excessive inflammation [2]. This balance is tightly regulated by a complex network of signaling pathways, cellular responses, and increasingly recognized metabolic processes. Metabolism is no longer viewed merely as a housekeeping function for energy production but as a dynamic source of signaling molecules that directly instruct immune cell function and inflammatory outcomes [3]. Furthermore, the metabolic state of both the host and the invading pathogen is now understood to play a decisive role in the nature and outcome of an infection [4].

Among the many metabolic immunomodulators, hydrogen sulfide (H_2_S) has recently emerged as a key gaseous transmitter with potent anti-inflammatory and cytoprotective properties [5].

In mammals, H_2_S is endogenously produced primarily via the transsulfuration pathway, catalyzed by the enzymes cystathionine β-synthase (CBS), cystathionine γ-lyase (CSE), and 3-mercaptopyruvate sulfurtransferase (3-MST) [6], and via a separate pathway involving thiosulfate sulfurtransferase (TST/TSTD1) [7,8,9], which is crucial for cyanide detoxification and mitochondrial sulfide oxidation. H_2_S influences a myriad of physiological processes, including vasodilation, neuromodulation, and mitochondrial bioenergetics [10]. Crucially, in the context of immunity, H_2_S has been shown to attenuate the production of pro-inflammatory cytokines, inhibit leukocyte adhesion, and scavenge reactive oxygen species (ROS), thereby protecting tissues from inflammatory damage. Conversely, dysregulation of H_2_S production is implicated in the pathogenesis of many chronic inflammatory diseases in humans [11].

Insects, diverse and ecologically dominant organisms, rely exclusively on innate immunity to defend against a wide array of microbial threats [12]. The fruit fly *Drosophila melanogaster* possesses an innate immune system that shares certain fundamental principles with mammalian immunity, including the use of conserved NF-κB signaling pathways (i.e., IMD and Toll pathways) and the production of antimicrobial peptides (AMPs) [13,14,15].

*Drosophila* also possesses orthologs of key sulfur metabolism enzymes—CBS and CSE—involved in the canonical transsulfuration pathway, as well as the *tst1* gene, which we recently described [16] and which encodes a cytoplasmic sulfurtransferase homologous to the human protein (TSTD1) [7,8], involved in cyanide detoxification and sulfide metabolism. In *Drosophila*, deletions in the genes encoding key enzymes of the H_2_S metabolic pathway (*cbs*, *cse*, and *tst1*) lead to H_2_S deficiency and hyperhomocysteinemia. These abnormalities induce oxidative stress and predispose the flies to chronic inflammation [16]. Knockouts of these three genes and their combinations allow us to analyze their distinct and overlapping roles in H_2_S metabolism across various aspects of the fly’s life, including basal metabolism, detoxification, and immune regulation.

Previous studies have shown that hemocyte activation and ROS production at wound sites protect flies against subsequent infection, a phenomenon interpreted as innate immune “training” [17]. Beyond the immediate immune response, a growing body of research has established that various adaptive cellular systems actively modulate immune function. For example, heat shock proteins (Hsps) are known to regulate inflammation and protect against proteotoxic stress during infection [18]. The JNK stress pathway, which integrates signals from ROS and inflammatory cues, controls the expression of detoxification enzymes and immune effectors [19]. Additionally, metabolic pathways such as insulin/IGF signaling play a critical role in redirecting energy resources toward immune defense in the case of infection [20].

However, the role of the H_2_S-producing system in these adaptive immune processes in *Drosophila* has not been investigated. Given the well-documented anti-inflammatory and cytoprotective properties of H_2_S, we hypothesized that genes involved in sulfur metabolism within the transsulfuration pathway play a key role in regulating innate immunity.

In this study, we investigated the immunological consequences of single, double, and triple knockout of H_2_S-producing genes following sterile thoracic injury and septic injury with *Bacillus subtilis* (*B. subtilis*).

The key question is whether immune dysregulation in the KO lines arises solely due to impaired H_2_S production and the associated accumulation of homocysteine, or whether it is also a consequence of the accumulation of toxic metabolites, e.g., cyanide, resulting from the *tst1* gene knockout.

In this investigation, we employ a multi-faceted approach, combining transcriptomics analysis, survival assays following sterile and septic injury of adult flies, and dynamic qRT-PCR profiling to corroborate our transcriptomic data. Our results show that H_2_S deficiency strongly activates the fly’s innate immune system. While KO of *tst1* per se does not significantly affect flies’ survival after septic injury, triple KO of the pertinent genes resulted in complex dysregulation of the innate immune system, significantly reducing fly survival after *B. subtilis* infection.

## 2. Materials and Methods

### 2.1. Drosophila Stocks

Transgenic knockout (KO) lines with deletions of the following genes: cystathionine β-synthase (*cbs*), cystathionine γ-lyase (*cse*), thiosulfate sulfurtransferase (*tst1*), as well as their combinations double (*cbs*; *cse*) and triple (*cbs*; *cse*; *tst1*) KO, have been previously described [16]. Experiments investigating the systemic immune response were conducted using 5-day-old virgin females of *D. melanogaster* from the control line 58492 (BDSC 58492, Bloomington, IA, USA) and transgenic lines with knockouts of sulfur metabolism genes. Line 58492 was selected as the control line, as all the knockout lines were derived from it using the CRISPR/Cas9 method, and its genetic background is the closest to that of the lines under investigation [16,21].

All flies were reared on standard sugar-yeast agar medium in a temperature- and humidity-controlled incubator at a constant temperature of 25 °C under a 12 h light/12 h dark (LD) cycle.

### 2.2. Flies Survival upon Bacterial Infection

To activate the systemic immune response, flies were infected with bacteria using the needle-pricking method [22,23]. The Gram-positive bacterium *Bacillus subtilis 168*, with a DAP-type peptidoglycan on its cell surface, was used. One day before the experiment, a single bacterial colony was cultured overnight in a sterile 10 mL tube containing 2 mL of LB medium in a thermoshaker at a temperature of 35 °C and a tube rotation speed of 200 revolutions per minute. The optical density of the bacterial solution was measured on a spectrophotometer at a wavelength of 600 nm. The bacteria, pelleted by centrifugation, were resuspended in PBS at a concentration of 5 OD. Five-day-old *D. melanogaster* females from the tested lines were briefly anesthetized with CO_2_ using a Benchtop Flowbuddy Fly Station (Genesee Scientific, Cat. No. 59-122BC, Morrisville, NC, USA). A 0.1 mm-diameter stainless steel needle (Fine Science Tools, catalog no. 26002-10, Foster City, CA, USA) was dipped into a bacterial suspension with a concentration of 5 OD and used to make a small puncture in the cuticle in the thorax region of a fly. In total, 20–25 flies were then transferred to new test tubes containing standard food. Individual flies that died on the day of the puncture due to severe injuries were not included in the subsequent analysis. For each infection, three to five replicate survival experiments were conducted. Eighty to ninety flies were used for each replicate. Survivors were counted daily, and flies were transferred to new vials with food every two days.

A clean puncture was performed using a needle sterilized with ethanol and washed with PBS. The median survival time was calculated over a period of 7 or 10 days, depending on the experimental conditions. Kaplan–Meier survival curves are presented for all experiments. The statistical significance of differences in survival time was assessed using the log-rank test with the Bonferroni correction. * *p* ≤ 0.05, ** *p* ≤ 0.01, *** *p* ≤ 0.001.

### 2.3. Quantification of Microbial Load for Growth Kinetics

Five-day-old female flies were infected with *B subtilis.* At defined time points after infection, flies were anesthetized, and three individuals were pooled per time point. Each fly pool was homogenized in 300 μL of LB medium (equivalent to 100 μL per fly) using an Eppendorf pestle driven by a cordless motor mixer (KIMBLE Pellet Pestle, Rockwood, TN, USA). The homogenates were serially diluted (10, 100, and 1000 times), and 50–100 μL aliquots were plated onto LB agar Petri dishes, followed by overnight incubation at 37 °C. At least three replicates of pooled flies were performed for each infection experiment, and a minimum of four independent biological replicates were performed in total. Colony-forming units (CFU) were counted manually to ensure accuracy. To verify proper bacterial growth, single-colony PCR was performed on randomly selected colonies using *B. subtilis*-specific primers [24]. To monitor the number of bacteria introduced during puncture, pooled fly samples were plated immediately after injection, allowing estimation of the initial dose of *B. subtilis* entering the fly during injection and to exclude variability associated with the injection technique. Statistical differences in bacterial load between groups were assessed using one-way analysis of variance (ANOVA). Fourth-order polynomial regression was used to fit bacterial load kinetic curves. Shaded areas in the graphs represent the 95% confidence intervals of the regression model, calculated based on the *t*-distribution.

### 2.4. Quantification of Pathogen Load upon Death (PLUD)

From a population of flies infected with *B. subtilis*, a single fly exhibiting pronounced symptoms of infectious pathology was selected: loss of locomotor activity, inability to move vertically, and lack of response to mechanical stimulation. Sample preparation and homogenate plating were performed in accordance with the protocol in the “Quantification of microbial load for growth kinetics” method. Results were expressed as CFU per fly [22,25]. For PLUD data, the median and interquartile range (Q1–Q3) are shown, as the distribution of values was characterized by marked asymmetry.

### 2.5. RNA Extraction

Total RNA was isolated using RNAzol RT reagent (Molecular Research Center, Cincinnati, OH, USA). Briefly, 10–20 flies were homogenized in 200 μL of RNAzol reagent, after which 80 μL of dH_2_O was added, and the mixture was incubated on ice for 10 min. The homogenate was then centrifuged at 15,000× *g* for 10 min at 4 °C. The resulting supernatant was collected and transferred to a new sterile 1.5 mL tube. An additional purification step was performed by adding 1 μL of 4-bromoanisole (MRC, BN 191). The mixture was incubated at room temperature for 3 min and centrifuged again at 15,000× *g* for 10 min at 4 °C. The supernatant was transferred to a new sterile tube, and 200 μL of isopropyl alcohol was added to precipitate the RNA. The mixture was incubated on ice for 30 min and then centrifuged at 20,000× *g* for 20 min at 4 °C. The RNA pellet was washed twice with chilled 75% ethanol and dissolved in RNase-free water.

RNA concentration was measured using a Qubit Fluorometer (Invitrogen, Carlsbrad, CA, USA). RNA quality was assessed using the RNA Nano Chips kit (cat#5067-1511, Agilent Technologies, Santa Clara, CA, USA) on an Agilent BioAnalyzer 2100.

### 2.6. RNA-Seq Library Preparation and Transcriptomic Analysis

RNA-seq libraries were prepared using the VAHTS Universal V8 RNA-seq Library Prep Kit for Illumina (cat# NR605-01, Vazyme Biotech, Nanjing, China) according to the manufacturer’s protocol. Sequencing was performed on an Illumina NextSeq 2000 platform. Raw reads obtained with the Illumina NextSeq 2000 device (Illumina, Inc., San Diego, CA, USA) were assessed for passing quality thresholds with fastqc, subsequently trimmed of adaptor sequences and low-quality reads with Trimmomatic software v0.40 [26], and aligned on a reference genome of *D. melanogaster* (BDGP v. 6.54) using the STAR aligner [27]. SAM files were converted to BAM, sorted and indexed with samtools v1.24 [28], and BAM files were subjected to the feature Counts utility to count exon-spanning reads on each gene [29]. Differentially expressed gene (DEG) lists were statistically determined with various functions implemented in the edgeR 3.36.0 package [30]. Gene selection was based primarily on passing of a false discovery rate-corrected *p*-value ≤ 0.05. Gene set enrichment analysis was conducted with the clusterProfiler 4.12.x package, and all gene expression-related visualization tasks were done with the help of the ggplot2 4.0.3 package [31]. RNA sequencing was performed using the equipment of the Engelhardt Institute of Molecular Biology RAS ‘Genome’ Centre (http://www.eimb.ru/rus/ckp/ccu_genome_c.php (accessed on 2 June 2025)). The sequence data were deposited in the NCBI GEO database under accession number GSE333980. The original RNA sequencing data presented in the study are openly available in the GEO database with accession number GSE333980.

### 2.7. Gene Expression via qRT-PCR

Total RNA was extracted from 10–15 flies per sample using RNAzol RT reagent (Molecular Research Center, USA) as described in Section 2.5. Three independent biological replicates were analyzed per genotype and condition. Reverse transcription was performed using MMLV reverse transcriptase (Evrogen, Moscow, Russia) with random hexamer primers [Random(dN)_10_]. Quantitative real-time PCR (qRT-PCR) was carried out on an Applied Biosystems 7500/7500 Fast Real-Time PCR System (Applied Biosystems, Foster City, CA, USA) in 96-well plates using qPCRmix-HS SYBR + LowROX (Evrogen, Russia). Each reaction was performed in technical triplicate. The thermal cycling protocol was as follows: initial denaturation at 95 °C for 3 min, followed by 40 cycles of 95 °C for 15 s, 60 °C for 20 s and 72 °C for 30 s. Melting curve analysis was performed to confirm the specificity of amplification. The relative expression of each gene was calculated using the 2^−ΔΔCt^ method, with *RpL32* as the reference gene for normalization. Data points represent the mean ± standard deviation from three independent biological replicates. To compare mRNA levels between the studied groups, one-way analysis of variance (ANOVA) followed by Tukey’s HSD post hoc test was used. *p*-values ≤ 0.05 were considered statistically significant. * *p* ≤ 0.05, ** *p* ≤ 0.01, *** *p* ≤ 0.001. The primers used in the qRT-PCR experiments are listed in Table S1.

## 3. Results

### 3.1. Knockout of Transsulfuration Pathway Genes Alters Survival and Bacterial Clearance After Septic Injury

To assess the role of genes involved in the transsulfuration pathway in flies’ innate immunity, survival experiments were conducted using the control 58492 line, as well as lines with single, double, and triple knockouts in the transsulfuration pathway genes.

**Figure 1:**
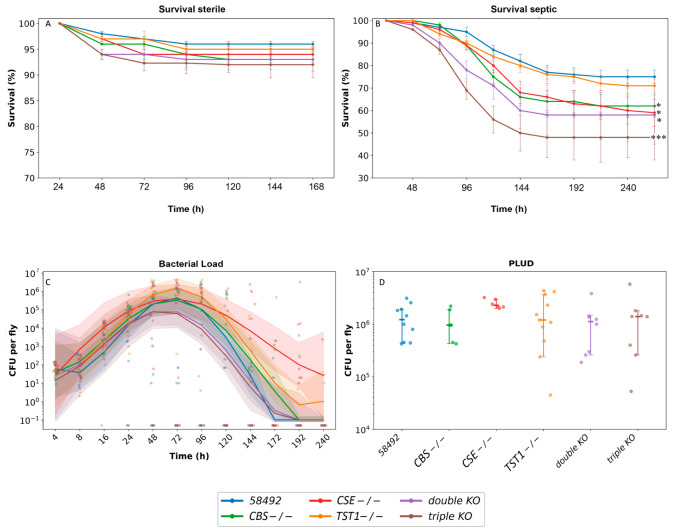
Survival and bacterial load kinetics in *Drosophila* lines with knockouts of transsulfuration pathway genes. (**A**) Survival kinetics following sterile injury. (**B**) Survival kinetics following septic injury with *B. subtilis*. The median survival time was calculated. To compare survival curves, the log-rank test was used with Bonferroni correction, * *p* ≤ 0.05, *** *p* ≤ 0.001 (KOs compared with the 58492 line). (**C**) Kinetics of *B. subtilis* load in the control line (58492) and lines with single and combined gene KOs (*cbs*, *cse*, *tst1*). Solid lines indicate a fourth-order polynomial regression. Shaded areas represent the 95% confidence intervals. All experiments were performed with *n* ≥ 3 independent biological replicates. Statistical significance was estimated using one-way ANOVA (*p* ≤ 0.05). (**D**) Measurement of pathogen load upon death (PLUD) following *B. subtilis* infection. Each data point represents a pooled sample from three flies. The differences are not statistically significant.

First, we analyzed the survival of flies after sterile injury (Figure 1A). Sterile injury under non-sterile conditions mimics the damage and contamination sustained by flies in their natural habitat and activates the immune response in *Drosophila.* Such injury may lead to improved immune protection for the fly, suggesting a form of innate immune “training” [17]. However, in flies with KOs of genes involved in the transsulfuration pathway, injury itself may lead to chronic inflammation. Our experiments monitoring the survival of flies after sterile injury failed to reveal significant differences between the lines compared. We can only speak of a trend toward slightly better survival rates in the control 58492 and *TST1-/-* lines compared to the other KOs (Figure 1A).

Then, we analyzed fly survival following septic injury with *B. subtilis.* Triple KO flies exhibited the lowest survival rate in these experiments, while the control (58492) and *TST1-/-* lines had the best survival rates (Figure 1B). Interestingly, the double KO line showed the greatest variation in survival rates, whereas the lines with knockouts of the *cse* and *tst1* genes were characterized by a longer period of mortality monitored among individual flies.

In parallel with our survival experiments, we examined the growth kinetics of the *B. subtilis* load in all studied lines. In the control line, the infection follows a classic kinetic curve (proliferation–peak–clearance) (Figure 1C): a phase of exponential *B. subtilis* proliferation is observed, reaching peak values (10^5^–10^6^ CFU) after 48–96 h. The mean value is 6 × 10^4^ CFU. The peak is followed by a phase of active clearance. Thus, after 120 h, the bacterial load decreases significantly, and a significant proportion of replicates falls below 100 CFU. This observation apparently indicates effective elimination of the pathogen and restoration of microbial homeostasis in the flies.

In lines with single KOs of transsulfuration pathway genes, more rapid bacterial proliferation is observed during the first 24 h. For example, in line 58492, 24 h after septic injury, the median bacterial load reaches 6 × 10^4^ CFU, whereas in lines with single knockouts, it ranges from 6 × 10^4^ to 5 × 10^5^ CFU. The main differences between the lines are observed 96–120 h after injury, when rapid bacterial elimination begins in the control line, completed by 172 h. In the line with the *cbs* gene knockout, bacterial elimination occurs more slowly and is completed only by 240 h. In the *CSE-/-* KO line, while the peak of bacterial proliferation is slightly shifted to the left, elimination slows down after 144 h, and even 240 h after septic injury, infected flies with a bacterial load of up to 10^3^ CFU remain at certain points. The *TST1-/-* line is characterized by the highest bacterial load (maximum 4 × 10^6^) and the greatest range of CFU values. In this line, infected flies with CFU counts ranging from 100 to 500 also remain 240 h after septic injury. It should be noted that for the *CSE-/-* and *TST1-/-* KO lines, which demonstrate delayed clearance of infection, a prolonged mortality trend is also observed, judging by the survival curve (Figure 1B).

Notably, the bacterial load curve for the double and triple knockout lines differs significantly from that of the control line. After 8 and 16 h, the curve is slightly shifted to the left; the peak for the triple and double KO lines occurs at 48 h. Although the maximum KOE level reaches 10^6^ at certain points, the average KOE value is 10^4^ (Figure 1C and Figure S1). In these lines, the active elimination phase begins after 72–96 h, which coincides with the peak in fly mortality (Figure 1B). Thus, on the one hand, an effective immune response prevents bacterial proliferation to the level observed in the control line (7 × 10^5^ KOE), while on the other hand, it leads to fly death at lower KOE values. Analysis of the bacterial load at the time of death (PLUD, Figure 1D) shows that the average lethal threshold for flies with single knockouts is 10^6^ KOE, which is comparable to the control. However, lines with combined KOs exhibit marked variability: some flies die at significantly lower values (10^4^–10^5^ KOE).

These results indicate that disruption of the transsulfuration pathway leads to pronounced instability of the innate immune response. In particular, many double and triple KO flies die during the peak of bacterial proliferation (48–72 h), earlier than flies of other genotypes (72–96 h). This early mortality cannot be explained by higher bacterial loads. Some double and triple KO flies die at lower bacterial burdens (10^4^–10^5^ CFU) compared to controls and single KOs (~10^6^ CFU), while the median lethal loads are broadly similar across genotypes. We therefore propose that in these flies, death may result not from uncontrolled bacterial growth, but from an excessive or dysregulated immune response. Although these data point to an immune-mediated pathology, they do not provide definitive proof of this assumption. Direct histopathological examination or measurement of inflammatory markers is required to confirm this interpretation.

### 3.2. Kinetics of Immune Gene Expression After Sterile and Septic Injury

**Figure 2:**
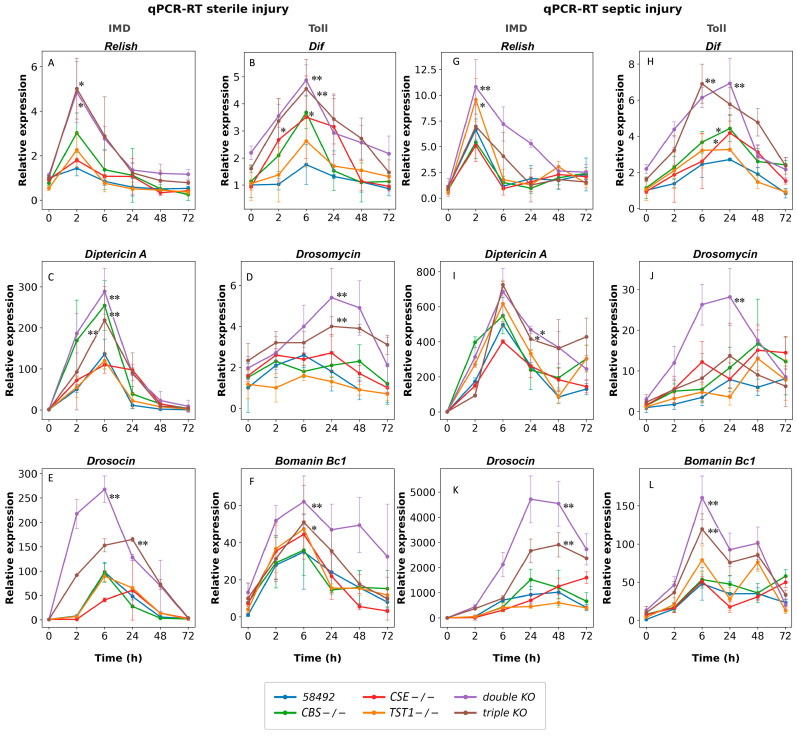
Kinetics of immune gene expression following sterile and septic injury in *Drosophila* lines with knockouts of the transsulfuration pathway genes. (**A**–**F**) Kinetics of relative expression of immune response genes (*Relish*, *Dif*, *Diptericin A*, *Drosomycin*, *Drosocin, Bomanin Bc1*) measured by qRT-PCR following sterile injury. (**G**–**L**) Kinetics of relative expression of immune response genes (*Relish*, *Dif*, *Diptericin A*, *Drosomycin*, *Drosocin*, *Bomanin Bc1*) measured by qRT-PCR following septic injury. Data represent the mean ± standard deviation from three biological replicates; * *p* ≤ 0.05, ** *p* ≤ 0.01, *t*-test. All experiments were performed with *n* ≥ 3 independent biological replicates.

To characterize the molecular response to injury, we analyzed the induction kinetics of key transcription factors and antimicrobial peptides (AMPs) by qRT-PCR. Sterile injury led to increased expression of key transcription factors involved in the IMD and Toll pathways (e.g., *Relish* and *Dif*) (Figure 2A,B), whose expression varies among KO lines. Notably, the highest induction was observed in double and triple KO lines, whereas the lowest induction was evident in the control line (58492) and the line with a *tst1* gene KO. qRT-PCR analysis of major AMPs revealed similar differences in expression patterns in the lines studied (Figure 2C–F).

Following septic injury, we observed a strong induction of the transcription factors *Relish* and *Dif*, and AMPs, including *Diptericin A*, *Drosomycin*, and *Bomanin Bc1*, with the greatest induction in double and triple KO flies (Figure 2G–L). Interestingly, *Drosomycin* was maximally induced only in the double KO, while showing similar expression levels in all other lines. The lowest induction was observed in the control and *TST1*^−^/^−^ lines.

### 3.3. Analysis of Transcriptomic Libraries: Hydrogen Sulfide Deficiency Promotes the Development of a Pro-Inflammatory Transcriptional Signature

Knockouts of sulfur metabolism genes, leading to reduced H_2_S levels and hyperhomocystenemia (primarily in *CBS-/-*, double, and triple KO lines), apparently cause oxidative stress in *Drosophila* [16], which may lead to chronic inflammatory processes.

To further investigate the consequences of these gene knockouts, we analyzed transcriptomic libraries obtained from 5-day-old females of the control 58492, *TST1-/-*, double, and triple KO lines.

**Figure 3:**
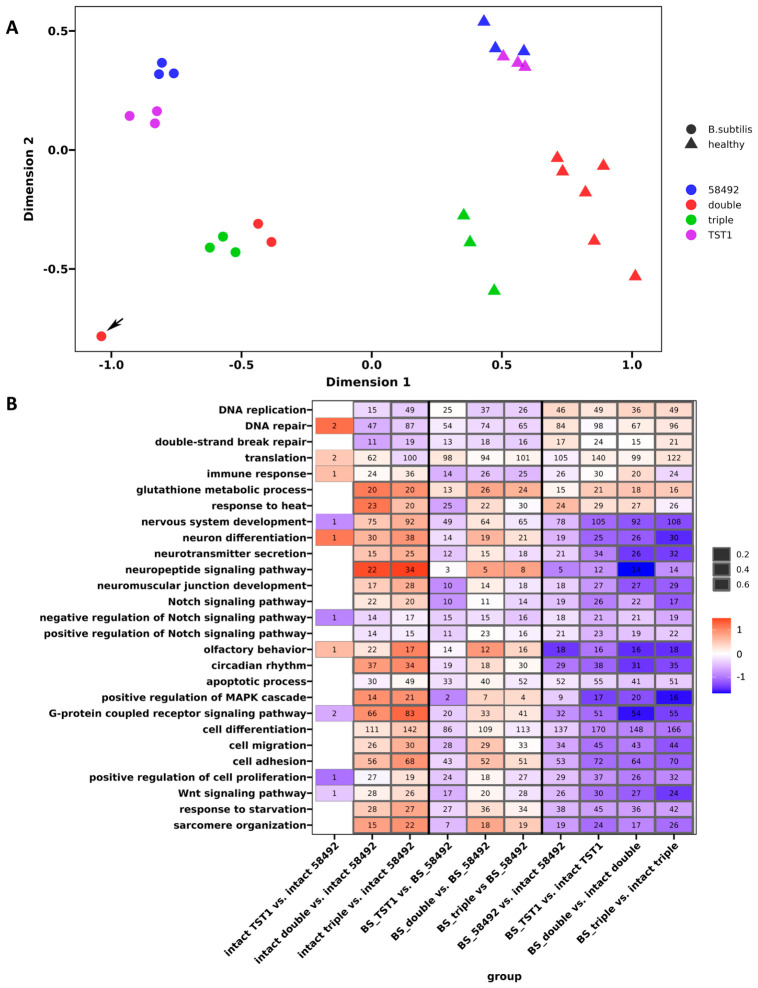
(**A**) Multi-dimensional scaling of log-scaled CPM expression. (**B**) GSEA analysis of differentially expressed genes in females with single *TST1-/-*, double, and triple KO. Three pairwise comparisons were done: 1. Intact (healthy) KO flies to control 58492 intact flies. 2. Infected with *B. subtilis* (B.S.) KO flies to infected 58492 flies. 3. *B. subtilis* infected flies to intact flies of the same genotype. Gene ontology (GO) categories were used as terms. The color of each cell represents the averaged LogFC value of genes enriched in the category (color code shown on the middle right side of the plot), which is also depicted by the number of genes in each cell. Grey border thickness represents the proportion of enriched genes, divided by the number of all genes in a category.

Here, we focused on the *TST1*^−^/^−^, double, and triple KO lines, excluding single *CBS-/-* and *CSE-/-* KOs. This choice was based on two observations from the survival and bacterial load experiments. First, single *CBS-/-* and *CSE-/-* KOs exhibited only mild phenotypes: they showed delayed bacterial clearance and prolonged mortality, but their survival rates and lethal bacterial loads (PLUD ~10^6^ CFU) remained comparable to the control line (Figure 1B,D). Consistent with this, qRT-PCR analysis revealed only moderate induction of immune genes in single *CBS-/-* and *CSE-/-* KOs compared to double and triple KOs (Figure 2A–L). Second, the most pronounced and distinct phenotypes—including high survival variability, death at low bacterial loads (10^4^–10^5^ CFU), and the greatest immune dysregulation—were observed in double and triple KOs (Figure 1B–D). The *TST1*^−^/^−^ single KO, while showing a mild phenotype, served as an essential control to reveal the contribution of *tst1* loss in the double KO background (triple KO). Thus, this combination of genotypes allowed us to capture the full spectrum from mild (*TST1*^−^/^−^) to severe (double KO) and decompensated (triple KO) immune-metabolic dysregulation. The flies were analyzed without injury and after septic injury with *B. subtilis* at the 6 h time point (Figure 3A,B). We chose the 6 h time point for transcriptome analysis because the most significant changes in the expression levels of most genes involved in the immune response are observed 4–12 h after injection [32]. The analysis was conducted to identify differences in the expression of genes involved in key immune response pathways (IMD and Toll), as well as in related processes such as protease regulation, opsonization, phagocytosis, digestion, and stress response.

Multi-dimensional scaling of log-scaled CPM expression values of all samples studied revealed clear separation between intact and *B. subtilis*-infected samples (Figure 3A), which is indicated on the Dimension 1 X-axis. Visible clustering of control non-infected flies with double and triple KOs is also evident, while the difference between intact control flies and *TST1*^−^/^−^ KO flies is relatively low. *B. subtilis*-infected samples are separated into two major groups: one includes control 58492 flies and flies with *tst1* deletion, and another includes double and triple KO flies. One of the infected double KO samples appeared as an outlier in the MDS plot (Figure 3A, indicated). This sample passed all standard quality control metrics (RNA integrity, read counts, alignment rate) and was retained in the analysis. The analysis demonstrated that removal of this sample did not qualitatively affect the differential expression results or the main conclusions (Figure S2).

A series of comparisons was conducted to determine the role of genes involved in sulfur metabolism in the immune response of *Drosophila*: Gene expression levels in KO lines were compared to the control line under normal conditions without any infection. Gene expression levels following septic injury were compared to the corresponding intact lines. Gene expression levels in KO lines following septic injury were compared to those of the control line after the same septic injury.

GSEA analysis (Figure 3B) shows that double and triple knockouts have a significant impact on all vital processes. Interestingly, when comparing infected flies (with double and triple KOs) to the infected control line 58492, a similar pattern of changes is observed, which differs significantly from the changes revealed in the infected *TST1-/-* line. It is also evident that *B. subtilis* infection induces expression of gene clusters involved in DNA repair, replication, and translation, as well as in the immune response, heat response, and glutathione metabolism. Notably, all other vital processes, including the development and functioning of the nervous system, as well as cell division and differentiation, are suppressed. Thus, when fighting infection, the fly’s organism apparently sacrifices growth and reproduction in favor of survival.

**Figure 4:**
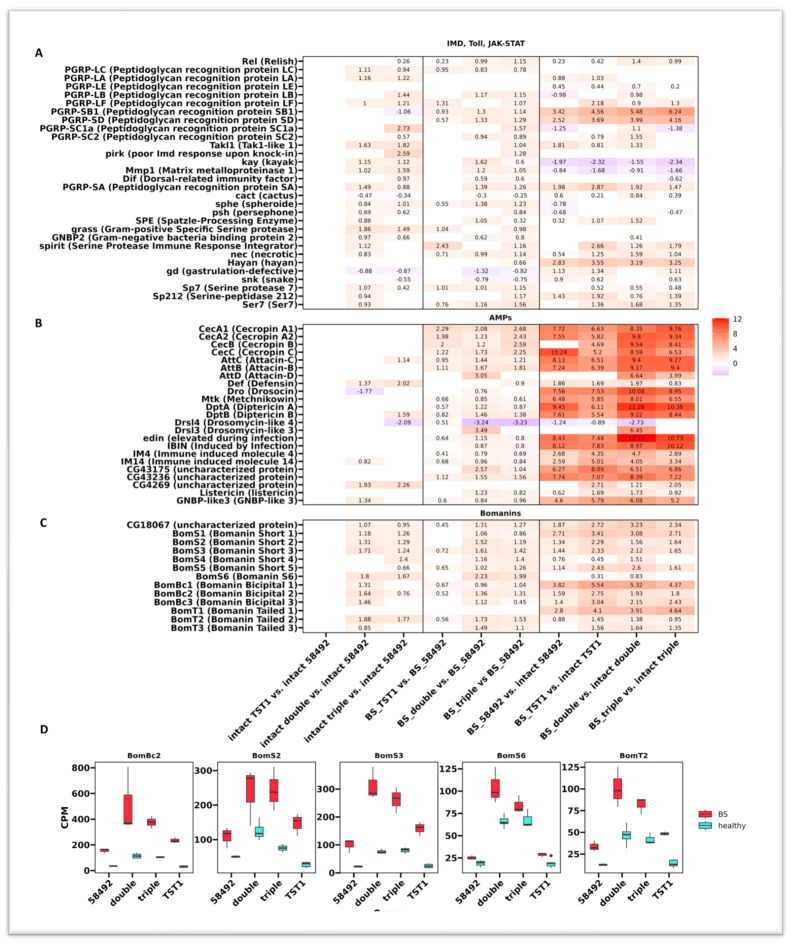
Differential expression of genes involved in immune response: Gene selection was based on passing a false discovery rate-corrected *p*-value < 0.05. (**A**) Components of IMD, Toll, and JAK-STAT pathways. (**B**) AMPs. (**C**) Bomanins in intact KO flies and after septic injury with *B. subtilis*. Three pairwise comparisons were made: 1. Intact (uninfected) KO flies to control 58492 intact flies. 2. Infected KO flies to infected 58492 flies. 3. Infected flies to intact flies of the same genotype. Heat maps display LogFC values for genes of interest, with blue representing genes with decreased expression (logFC < 0) and red representing genes with increased expression (logFC > 0). (**D**) Box plots displaying the expression levels of Bomanin cluster genes under control conditions and after septic injury. The x-axis represents the control line (58492), a single *tst1* gene deletion (TST1), and multiple gene knockouts (double and triple). Color coding corresponds to injected *B. subtilis* (red rectangles) and uninfected flies (blue rectangles). In the box plots, the center line represents the median, the rectangle represents the interquartile range (IQR), and the whiskers represent the minimum and maximum values. Expression levels are presented as normalized values per million. All genes in the box plots have significant differential expression (FDR ≤ 0.05) in at least one of the comparisons used in Figure 4C.

Interestingly, under basal conditions, lines with double and triple KOs exhibited increased expression of several key genes involved in immune response. In particular, we detected elevated levels of transcripts encoding the peptidoglycan-recognizing proteins (PGRPs) PGRP-LC, PGRP-LA, and PGRP-LF (Figure 4A). Notably, PGRP-LC and PGRP-LA are primary sensors of bacterial infection [33], whereas PGRP-LF acts as a negative regulator of the IMD pathway [34]. In addition, increased expression of *PGRP-LB*, an amidase that degrades peptidoglycan and limits activation of the IMD pathway, was observed predominantly in the triple KO line. Furthermore, the triple KO line exhibited elevated basal expression of *pirk* (PGRP-LC-interacting protein), a well-characterized negative feedback regulator of the IMD pathway. Pirk functions by binding to PGRP-LC and preventing its interaction with downstream signaling components, thereby attenuating NF-κB activation [35].

Despite the transcriptional activation of both stimulatory and inhibitory components, no change in the expression of most AMPs (except *def*) was observed (Figure 4B), probably indicating the presence of compensatory mechanisms that prevent the development of uncontrolled autoimmune inflammation under control conditions.

Following septic injury, all lines, particularly those with double and triple KOs, exhibited an active immune profile (Figure 4 and Figure S3). Thus, in this series of experiments, strong *Relish* induction is observed primarily in double and triple KO lines (Figure 2G). In contrast, the intracellular receptor *PGRP-LA* was induced mainly in control and *TST1-/-* lines. Notably, the intracellular receptor *PGRP-LE* was induced in all lines. This receptor, together with PGRP-LC, increases sensitivity to bacterial signals [36]. Additionally, *PGRP-SD* and *PGRP-SB1* were induced in all lines, with the strongest upregulation observed in KO lines, especially in double and triple KOs. PGRP-SD transmits a signal to the PGRP-LC/SA receptors [37], whereas PGRP-SB1 cleaves DAP-containing peptides due to its amidase activity but does not inhibit the IMD pathway [38].

At the same time, negative regulators were induced primarily in double KO flies, including the zinc-dependent amidase *PGRP-SC2* and the immunomodulatory cytokine *Diedel* (See box plots in Figure S3) [39,40,41]. Furthermore, in double and triple KO lines under control conditions, we observed basal activation of the stress JNK pathway. This conclusion is based on increased transcript levels of the key JNK pathway components: *Tak1* (a central mediator activating both NF-κB and JNK), the transcription factor *kayak* [42,43], and the downstream JNK target *Mmp* [44].

As for the Toll pathway (Figure 4), the expression levels of the transcription factors *Dif/Dorsal* and the inhibitor *cactus* in intact KO flies remained largely unchanged. At the same time, in double, and to a lesser extent, in triple KO lines, increased expression of several genes involved in the Toll pathway was observed, including *PGRP-SA*, *GNBP2*, and Toll cascade serine proteases (*psh*, *Grass*, *SPE*, *Sphe*, *spirit*). In contrast, the expression of the serine proteases *gastrulation defective* (*gd*) and *snake* (*snk*) was reduced both under control conditions and in infected double and triple KOs (Figure 4 and Figure S3). These proteases, originally characterized as participants in the dorso-ventral organization of the embryo, are also involved in the adult immune response within the Toll pathway [15]. The observed decrease in their expression indicates that normal functioning of the transsulfuration pathway is necessary to maintain basal expression and/or stability of this specific proteolytic branch of the Toll pathway.

A negative regulator of the Toll pathway, the serine protease *Necrotic* (*nec*), was expressed at higher levels in double KO flies compared to the control line. Necrotic functions by inhibiting the proteolytic cascade, which activates Spätzle, thereby limiting the intensity and duration of the Toll-dependent immune response [45]. Its upregulation in double KO flies likely represents a compensatory feedback mechanism to restrain excessive Toll pathway activation caused by H_2_S deficiency. Notably, after septic injury, *nec* expression was increased in all lines, more strongly in KOs, suggesting a general stress-induced enhancement of this negative regulatory loop (Figure S3).

Following septic injury, all lines showed induction of Toll pathway components; however, this induction was stronger in KO flies compared to the control line. Specifically, the highest levels were observed for the following genes: the transcription factor *Dif* (Figure 2), *GNBP-like3* [46], and the serine protease *Hayan* (Figure 4). This protease is a key regulator of innate immunity, which mediates the pro-phenoloxidase (PPO) activation cascade and the Toll signaling pathway [47]. The levels of serine proteases *spirit*, *Ser7* [48,49], and *Sp7*, which is involved in bacterial clearance [50], were also upregulated (Figure 4 and Figure S3). In addition, *SPE* (Spätzle-processing enzyme) expression was elevated, particularly in the *TST1^−^/^−^* and double KO lines, and to a lesser extent in triple KO flies.

When infected KO lines were compared to the infected control line, triple KOs showed the most pronounced differences in expression levels of IMD and Toll pathway genes, affecting both positive and negative regulators. In the case of a double knockout, these differences were less pronounced, with PGRP-SC2 being the only negative regulator in which changes in expression were observed. It is of note that the *TST1-/-* single KO exhibited only minimal changes (Figure 4A). This graded response—triple > double > *TST1-/-* to control—demonstrates that the extent of transcriptional immune dysregulation correlates with the degree of H_2_S pathway disruption. The triple KO flies, lacking both transsulfuration and sulfide metabolism genes, display the most pronounced alterations in the expression of both positive and negative regulators, consistent with their poorest survival outcome.

### 3.4. Elevated Baseline Levels of Bomanins and Marked Induction of AMPs Expression Following Septic Injury in H_2_S-Deficient Flies

To assess the functional consequences of the observed activation of the immune pathway, we analyzed the expression levels of key antimicrobial peptides (AMPs) and the body’s defense factors—bomanins—under normal conditions and following septic injury induced by *B. subtilis* (Figure 4B,C). Under control conditions, double and triple KO flies exhibited increased basal expression of the AMP *Defensin* and most *Bomanin*-encoding genes, including *BomS1, BomS2*, *BomS3*, *BomS6*, *BomT2*, and *BomBc2* (Figure 4C,D). All these cysteine-rich peptides contain a specific pattern of cysteine residues that form internal disulfide bridges [49,50,51]. This structural feature enables their interaction with sphingolipids such as glucosylceramide, which are critical components of fungal membranes and host lipid rafts. Bomanins have been shown to play a role in toxin neutralization. Notably, *BomS6*—which is critically important for survival upon exposure to the neurotoxin verruculogen [51,52,53]—and *BomT2* demonstrated increased expression levels in double and triple KOs under control conditions (Figure 4D). We speculate that elevated basal levels of *Defensin* and *Bomanins* serve as a specific biomarker of a neuroprotective response against endogenous intoxication caused by oxidative stress in KO flies lacking H_2_S-producing enzymes.

Following septic injury, a strong induction of antimicrobial peptides (AMPs) was observed, with the highest degree of induction noted in flies with double and triple knockouts, and the lowest in flies of the *TST1-/-* line. Only *Attacin D* (*AttD*) was virtually not induced in the control and *TST1-/-* lines. AttD is an atypical effector of the IMD pathway. Recent studies have revealed its unique role in mediating inflammatory damage to the Malpighian tubules, where it remains within the tubular cells and causes damage as a result of its aggregation and oligomerization [54]. The greatest post-infection induction in double and triple KOs was observed for *Edin*, *Drosocin*, *Diptericin A*, *Diptericin B*, *Cecropin A2*, and *Cecropin B*, *Attacin B*, and *Attacin C*. Interestingly, *Drsl3* was induced only in the double KO line.

As expected, *Bomanin* levels were upregulated after septic injury in all lines, with particularly strong induction detected in double and triple KO flies. The most significantly upregulated genes in this category were *Bombardier*, *BomBc1*, *BomBc3*, *BomS1*, *BomT1*, *Dso1* (*Im4*), and *Dso2* (*Im14*) (Figure 4C,D).

### 3.5. Compensatory Activation of Serpins and Reprogramming of Lectin and Phagocytic Receptor Patterns in Knockout Lines

**Figure 5:**
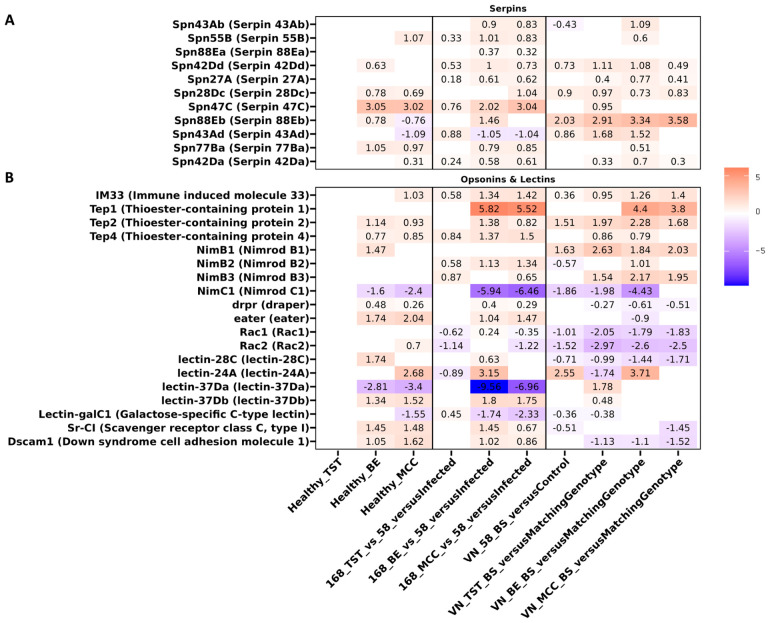
Differential expression of genes encoding: (**A**) serpins, (**B**) opsonins, and lectins in intact KO flies and after septic injury with *B. subtilis*. Three pairwise comparisons were done: 1. Intact (healthy) KO flies to control 58492 intact flies. 2. Infected *B. subtilis* (BS) KO flies to infected *B. subtilis* 58492 flies. 3. *B. subtilis* infected flies to intact flies of the same genotype. The heatmaps display the logFC values for the genes of interest: blue indicates genes with reduced expression (logFC < 0), while red indicates genes with increased expression (logFC > 0).

Serine proteases and their inhibitors (serpins) are involved in innate immunity and morphogenesis [48], controlling proteolytic cascades and rapid physiological responses (Figure 5). To this end, several *Drosophila* serpins function in immune responses (i.e., *Spn43Ac*, *Spn27A*, *Spn28Dc*, *Spn42Dc*, *Spn77Ba*) [48,55]. Moreover, *Spn47C*, *Spn27A*, *Spn28Dc*, *Spn77Bc*, and *Spn55B* are strongly upregulated in response to toxic α-synuclein accumulation [56] and are induced in starved larvae [57], indicating their activation under stress.

Under control conditions, we observed elevated expression of several serpins, i.e., *Spn47C*, *Spn77Ba*, and *Spn28D*, in double and triple KOs. *Spn77Ba* is the main inhibitor of proteases that trigger the melanization process, protecting tracheal and intestinal epithelia from damage and systemic inflammation [13,58]. After septic injury in all lines, *Spn28DC*, *Spn42Da*, and *Spn88Eb* were upregulated (Figure 5A and Figure S4). In infected double and triple KOs, the elevated level for most of the analyzed serpins (except *Spn43Ad*) [48,59] was also observed. The increased expression of these serpins in knockout lines, which exhibit heightened pro-inflammatory responses, likely indicates a compensatory mechanism aimed at reducing the intensity of the immune response.

Besides the pattern recognition receptors (PRRs), insect genomes encode secreted recognition molecules such as thioester-containing proteins (TEPs). These proteins play an important role in the host’s innate immune response by recognizing and promoting the elimination of various invading microbes [60]. TEPs represent secreted recognition molecules similar to mammalian complement C3/α2-macroglobulin [61]. In *Drosophila*, TEP2 and TEP4 promote phagocytosis of Gram-negative bacteria and fungi [62]. It is of note that *Tep* genes are induced by bacterial, fungal, and parasitic challenges [63,64].

Under control conditions, *Tep2* and *Tep4* expression levels were slightly elevated in double and triple KO lines (Figure 5B). This may reflect regulation of basal TEP expression by the JNK pathway, which is activated by oxidative stress in double and triple KOs [65,66]. After septic injury, *Tep1* was upregulated only in double and triple KO, *Tep4* in *TST1-/-* and double KO, and *Tep2* in all lines (more strongly in double KO) (Figure S5). *Tep1* and *Tep4* are among genes activated immediately after tissue damage [67], and *Tep4* modulates the immune response after infection [67] under JNK cascade control. The observed interline differences suggest an important immunomodulatory role of H_2_S in regulating the JNK stress response pathway.

Nimrod B proteins (NimB1–B5) represent secreted opsonins produced by hemocytes that bind bacteria [68] and apoptotic cells, enhancing efferocytosis [69]. We observed increased expression of the *NimB1* gene under control conditions in the double KO. Following septic injury, *NimB1* was induced in all lines, *NimB2* only in the double KO, while *NimB3* was upregulated in all KO lines.

Insects recognize pathogens that have entered the body using various pattern recognition receptors (PRRs), which identify pathogen-associated molecular patterns (PAMPs) on the surface of microorganisms [70]. Scavenger receptors (SCRs) and C-type lectins (CTLs) belong to the PRR family. We observed complex regulation of lectins and scavenger receptors depending on the status of the transsulfuration pathway genes (*cbs*, *cse*, *tst1*). The class C scavenger receptor (dSR-CI) and Dscam act as pattern recognition molecules that promote phagocytosis of both Escherichia coli and Staphylococcus aureus, but not yeast [71]. Notably, Dscam may function as either a phagocytic receptor or as an opsonin [72]. In our libraries, under control conditions, the expression of *dSR-CI* and *Dscam*—as well as in infected flies—was highest in flies with triple and double KOs. The observed constitutive upregulation of these receptors suggests that H_2_S deficiency mimics a state of “chronic infection” (Figure 5).

Proteins of the Nimrod family (Eater and NimC1) are transmembrane pattern recognition receptors. They are the primary phagocytic receptors for Gram-positive bacteria. Both proteins synergistically promote bacterial phagocytosis [73]. However, phagocytosis of microorganisms occurs even in the absence of NimC1, but not in the absence of Eater. Furthermore, Eater functions as a cell adhesion molecule [74]. *Eater* and *NimC1* were differentially expressed in the lines studied. Thus, *NimC1* expression was higher in the control line and the *TST1-/-* line, whereas *Eater* expression was elevated in the double and triple knockout lines under control conditions. Characteristically, following septic injury, the expression of both genes decreases (Figure S5).

Closely related secreted lectins 37Da and 37Db exhibited diametrically opposite expression patterns in the studied lines. This phenomenon mirrors the inverse regulation of the phagocytic receptors *Eater* and *NimC1* in response to H_2_S deficiency. Thus, in control and *TST1-/-* flies, *Lectin-37Da* is expressed, while in double and triple KOs, it is suppressed, and *Lectin-37Db* becomes strongly upregulated. This switch between isoforms indicates a reprogramming of both humoral pattern recognition and the repertoire of phagocytic receptors under conditions of H_2_S deficiency, which is characteristic of knockout lines.

### 3.6. Changes in the Transcription of Genes Involved in Digestion and Metabolism in Knockout Lines

Since the insect gut serves not only as a digestive organ but also as a key component of the innate immune system, acting as the first line of defense against foodborne pathogens [75], we investigated whether there are differences in the expression levels of key enzymes involved in digestion among the lines studied.

**Figure 6:**
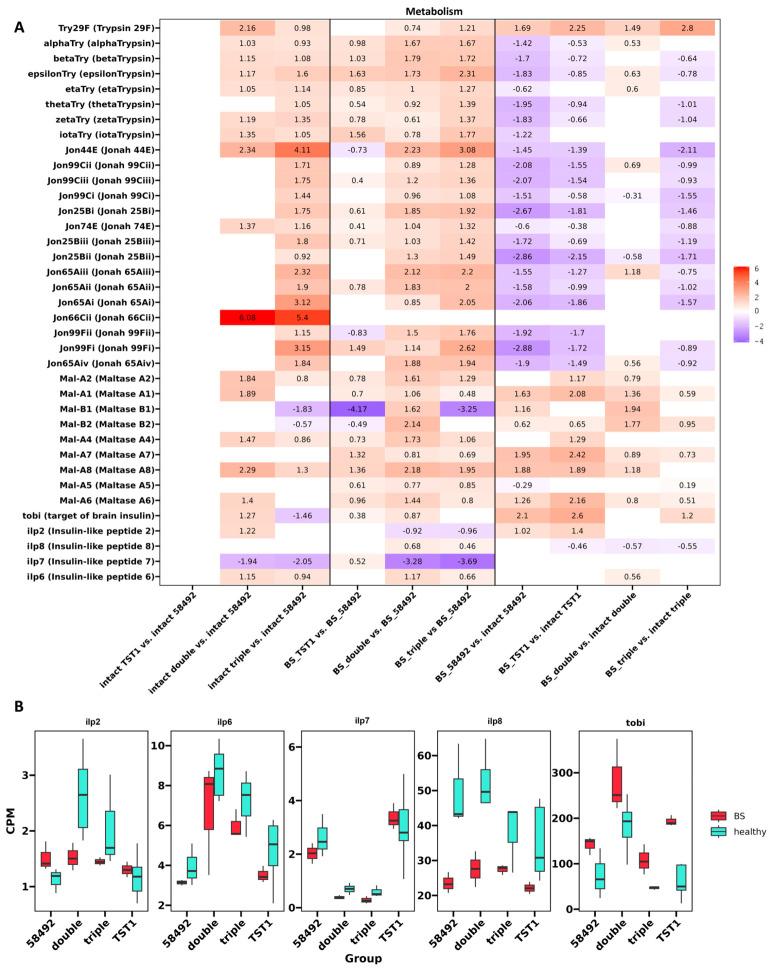
Differential expression of genes encoding digestive enzymes and insulin-like peptides. (**A**) Heat map showing expression of trypsins, Jonah proteases, and maltases in control and KO lines under control conditions and after septic injury. Three pairwise comparisons were done: 1. Intact (healthy) KO flies to control 58492 intact flies. 2. Infected *B. subtilis* (BS) KO flies to infected *B. subtilis* 58492 flies. 3. *B. subtilis* infected flies to intact flies of the same genotype. Heat maps display LogFC values for genes of interest, with blue representing genes with decreased expression (logFC < 0) and red representing genes with increased expression (logFC > 0). (**B**) Box plots illustrating differences in the expression of insulin-like peptides (*ilp2*, *ilp6*, *ilp7*, *ilp8*) across genotypes under control conditions and after septic injury. The central line represents the median, the box represents the interquartile range (IQR), and the whiskers represent the minimum and maximum values. Expression levels are presented in normalized counts per million. All genes in boxplots are significantly differentially expressed (FDR < 0.05) in at least one comparison from comparisons used in Figure 6A.

Under control conditions, double and triple KO lines showed increased expression of trypsin-encoding genes (alpha–zeta) active in different regions of the midgut [76] (Figure 6). A similar but distinct expression pattern was observed for the *Jonah* gene family, which encodes chymotrypsin-like serine proteases. These genes are normally expressed in the midgut and participate in digestion, but they are also known to be involved in immune responses [77,78]. In double and triple KO lines, we detected increased expression of several *Jonah* genes, including *Jon66Cii*, *Jon44E*, and *Jon74E*. In the triple knockout line, additional members of the *Jonah* family were also upregulated, namely *Jon99Ci*, *Jon99Cii*, *Jon99Ciii*, *Jon99Fi*, *Jon25Bi*, *Jon25Bii*, *Jon65Ai*, *Jon65Aii*, *Jon65Aiii*, and *Jon65Aiv* (Figure 6). After septic injury, the greatest reduction in the expression levels of both trypsin-encoding genes and Jonah proteases was observed in the control line, and to a lesser extent in *TST1*-/- and triple KO lines. Notably, in the double KO line, no reduction in trypsin or Jonah expression was detected after septic injury— their levels remained similar to basal conditions (Figure 6). At the same time, the relative expression levels of these genes in the infected double and triple knockout lines become upregulated in comparison with the infected 58492 line (Figure 6), suggesting that there is a certain balance in the expression levels of digestive proteases during the immune response.

It has been shown that septic injury triggers a robust innate immune response associated with significant energy expenditure and physiological reorganization [79]. Under control conditions, double KO flies exhibited elevated expression of several maltases (*Mal-A1*, *Mal-A2*, *Mal-A4*, *Mal-A6*, *Mal-A8*) and the gene *tobi* (target of brain insulin) (Figure 6). In contrast, triple KO flies showed the lowest *tobi* expression levels and only a modest increase in *Mal-A2*, *Mal-A4*, and *Mal-A8* expression. The gene *tobi* encodes α-glucosidase—a key component of insulin and glucagon signaling in the brain, playing an important role in glucose metabolism and energy balance [80].

Basal levels of *ilp2* and *ilp6* were also elevated in double and, to a lesser extent, in triple KOs, while *ilp7* (required for reproduction) was reduced. This suggests that oxidative stress and associated energy deficit in double KO trigger several adaptive mechanisms [81] involving activation of specific genes for carbohydrate mobilization. Thus, double KO flies show a clear transcriptional signature of carbohydrate mobilization, whereas triple KO flies largely lack this response, further highlighting the distinct metabolic phenotypes of these genotypes.

Notably, after septic injury, maltase expression was increased in all lines, with the highest induction observed in the *TST1^−^/^−^* KO line. Although the expression of *MalA2*, *A6*, *A8*, and *A7* was induced in the control and *TST1-/-* lines, in the infected KO lines, maltase expression levels remained higher than in the control infected line, except for *MalB1* and *MalB2*, whose expression was reduced in both *TST1-/-* and triple KO lines. Notably, after septic injury, all lines showed increased expression of the *tobi* gene, which promotes the release of sugar reserves to support the immune response. However, the brain’s insulin signal (*ilp2*) responded oppositely: its level remained unchanged in control flies and flies with the *TST1-/-* KO, but decreased in flies with double and triple KOs (Figure 6B). Consequently, *ilp2* levels became similarly low in all infected lines. This suggests that infection overrides genotype-specific differences, pushing all flies toward a common ‘low insulin’ state. Characteristically, in all lines, *ilp8* level was reduced after septic injury. This downregulation is typically associated with Toll pathway activation, which counteracts insulin signaling by redirecting nutrients (lipids and sugars) toward AMPs synthesis [82]. In contrast, *ilp6* levels remained comparatively high in double and triple KOs after septic injury, suggesting persistent insulin-like signaling from the fat body in H_2_S-deficient flies.

### 3.7. Chronic Oxidative Stress Activates Detoxification Systems and Stress Response in Double and Triple Knockout Lines

**Figure 7:**
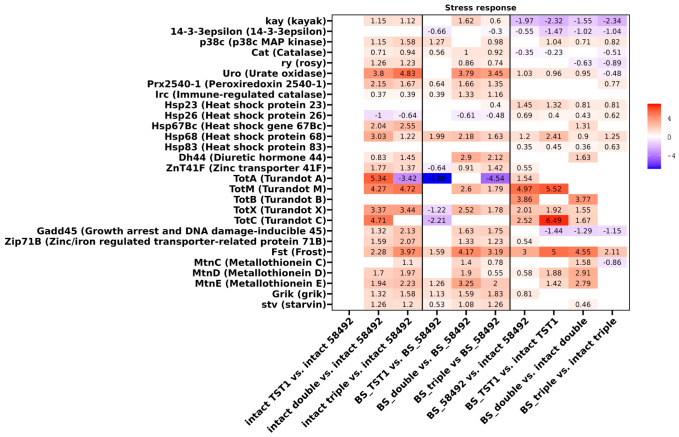
Differential expression of stress response genes in control and KO lines under control conditions and after septic injury. Three pairwise comparisons were done: 1. Intact (healthy) KO flies to control 58492 intact flies. 2. Infected *B. subtilis* (BS) KO flies to infected *B. subtilis* 58492 flies. 3. *B. subtilis* infected flies to intact flies of the same genotype. Heat maps display LogFC values for genes of interest, with blue representing genes with decreased expression (logFC < 0) and red representing genes with increased expression (logFC > 0).

The transcriptomic analysis carried out indicates a state of chronic systemic stress caused by impaired H_2_S metabolism in double and triple KO flies. This is evidenced by the activation of various detoxification systems, including increased expression of Cytochrome P450 genes, glutathione S-transferases (GSTs), UDP-glycosyltransferases (UGTs), and ecdysteroid 22-kinases [83] (Figure S6). After septic injury, several of these genes were further upregulated, including *Cyp6a8*, *Cyp6w*, *Cyp4ac3*, *Cyp6a18*, *Cyp309a2*, *Cyp12d1-p*, *Cyp6a23*, *Cyp4p3*, *Cyp309a1*, and *Cyp6a20*. In contrast, other genes were induced mainly in the control and *TST1-/-* lines: *Cyp4e3*, *CG14245*, *CG11878*, *CG6908*, *GstD2*, *GstD3*, *Ugt36A1*, *Ugt37C2* (Figure S6). Notably, *Cyp6a8* and *GstD5* were induced only in double KO; *GstD8* in double and triple KOs. Their increased expression following a bacterial infection suggests that they are essential for neutralizing toxins, alongside the standard production of antimicrobial peptides (AMPs). In addition to detoxification enzymes, double and triple KOs under control conditions exhibited elevated expression of genes important for stress response, including *hsp67Bc*, *hsp68*, *totm*, *totx*, *Gadd45*, *Fst*, *MtnD*, *MtnE*, *Grik*, *stv*, *Dh44*, *ZnT41F*, *Zip71B*, and (only in double KO) *totA* and *totC*. Among other stress-inducible genes, *Frost* expression was elevated under control conditions and induced by septic injury in all lines. *Metallothioneins* and *Diuretic hormone 44* were induced predominantly in double KOs (Figure 7).

Taken together, these data indicate that flies with H_2_S deficiency—particularly those with double and triple knockouts—exhibit a pronounced transcriptional response to stress, accompanied by elevated levels of detoxification enzymes, heat shock proteins, and metallothioneins, suggesting a state of chronic oxidative stress and metabolic adaptation.

After septic injury, *hsp23* and *hsp68* were induced in all lines. However, in transcriptomic libraries at 6 h post-infection, we did not observe significant induction of the major heat shock protein *hsp70*. Previously, we demonstrated complex interactions between the Hsps system and genes involved in H_2_S synthesis and metabolism in flies [84]. Since even sterile injury induces ROS production [17], we monitored *hsp70* expression by qRT-PCR in KO lines after both sterile and septic injuries.

**Figure 8:**
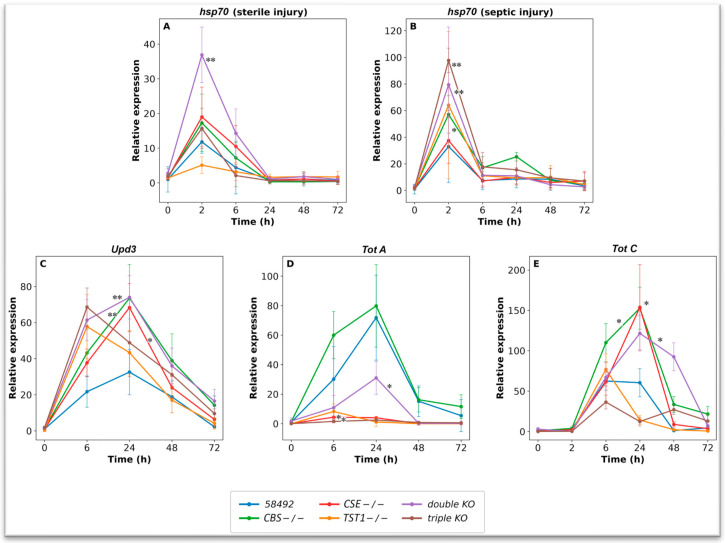
Kinetics of the relative expression of *hsp70*, *Upd3*, *TotA*, and *TotC* genes measured by qRT-PCR following injury. Data represent the mean ± standard deviation from three biological replicates. * *p* ≤ 0.05, ** *p* ≤ 0.01, *t*-test.

Our experiments demonstrated that cuticle damage per se induced *hsp70* transcription in all studied lines. Notably, a significant increase in *hsp70* induction after septic injury compared to sterile injection occurred preferentially in KO lines. In both cases, induction peaked at 2 h post-injury. Sterile injury caused maximal *hsp70* induction in the double KO line, whereas septic injury caused maximal *hsp70* induction in both double and triple KOs (Figure 8). These data suggest that basic proteostasis is severely disrupted in KO flies. Even minor damage to the cuticle leads to the formation of a greater number of damaged or aggregated proteins, which appears to trigger a stronger signal for the activation of HSF1—the primary regulator of proteotoxic stress and an inducer of Hsps. During infection, oxidative stress intensifies, leading to even stronger *hsp70* induction in double/triple KOs.

Among other stress-inducible genes regulated by the JAK/STAT pathway are genes of the *Turandot* family. In *Drosophila*, this family consists of eight genes whose expression is altered under various stress conditions, including infection [81]. The proteins of this family protect the epithelial cells of the tracheal system from the damaging effects of AMPs [83]. According to our transcriptomic analysis data, obtained 6 h after infection, the *TotA* gene was induced only in the control line, whereas the *TotC* gene was induced in all lines, with the highest levels observed in the control line and the double knockout line (Figure 7). Since *Turandot* genes typically show maximum expression after 16–24 h of infection [85,86], we performed qRT-PCR to examine the dynamics of *TotA* and *TotC expression* in our experiments.

The analysis demonstrated that *TotA* was induced mainly in control, *CBS-/-* KO flies, and to a lesser extent in double KO flies. On the other hand, *TotC* was induced in all lines, peaking at 24 h in *CBS-/-*, *CSE-/-*, and double KO lines, but was minimally expressed in triple KO (Figure 8).

After septic injury, *TotM* was induced in control and *TST1-/-* lines, and *TotX* in control, *TST1-/-*, and double KO (Figure 7). Thus, in the triple KO, we did not observe a significant increase in the expression levels of any genes belonging to the Turandot family after septic injury. This may account for the maximal compensatory *hsp70* induction seen in this line [86].

Turandot genes (*TotA*, *TotC*, *TotM*) are controlled by the JAK/STAT pathway and are activated by the cytokine-like ligand *Unpaired 3* (*Upd3*), as well as by the IMD pathway [87]. *Upd3* is normally expressed at very low levels and was not detected in our transcriptomic analysis; therefore, we examined its expression dynamics by qRT-PCR. Experiments have shown that *Upd3* expression was maximally induced in double and triple knockout lines and, consequently, cannot account for the differences in *TotA/TotC* expression observed in the lines under investigation. Taken together, these data indicate that triple KO flies fail to mount a proper Turandot response upon infection, which probably contributes to their severe proteostatic imbalance, judging by the highest *hsp70* induction. This observation highlights the critical role of the JAK/STAT-Turandot axis in mitigating stress-induced damage under conditions of H_2_S deficiency.

## 4. Discussion

This study demonstrates that a genetic disruption of sulfur metabolism—specifically, combined deletions of the *cbs*, *cse*, and *tst1* genes in *D. melanogaster* significantly alters the homeostasis of the whole innate immune system, leading to chronic inflammation, immune dysregulation, and altered survival rates following septic injury. The accumulated results indicate that the transsulfuration pathway and endogenous H_2_S production are key modulators of immune signaling, metabolic reprogramming, and stress responses in the intact organism. Transcriptomic analysis was carried out at a single time point (6 h after infection), while quantitative real-time PCR was used to detect dynamic changes in key immune response genes over a period of 2 to 72 h. Thus, the transcriptomic data should be regarded as providing an overall picture of key differences between lines, while the real-time PCR data provided temporal validation of key immune responses. Furthermore, as with any genetic study of Drosophila, the possibility of confounding factors cannot be entirely ruled out; however, the use of a single common genetic background (58492) for all knockout lines minimizes this risk.

### 4.1. Survival and Bacterial Load: Genotype-Specific Outcomes

Survival experiments following septic injury with *B. subtilis* revealed genotype-dependent differences. The control line (58492) and the *TST1-/-* single KO exhibited the best survival rates, whereas the triple KO showed the poorest survival. The double KO displayed the greatest variation in survival outcomes, suggesting an unstable immune–metabolic equilibrium.

Analysis of bacterial load kinetics provided mechanistic insights into these survival patterns. The control line exhibited a classic infectious curve: exponential bacterial proliferation peaking at 48–96 h (median ~6 × 10^4^ CFU), followed by active clearance and restoration of microbial homeostasis by 172 h. The triple KO displayed an atypical phenotype: bacterial proliferation was partially suppressed (peak median ~10^4^ CFU, lower than controls), yet some flies died at significantly lower bacterial loads (10^4^–10^5^ CFU compared to ~10^6^ CFU in controls).

The double KO showed intermediate characteristics: bacterial load peaked at 48 h, and active elimination began 72–96 h later, coinciding with a peak of fly mortality. Analysis of bacterial load at the time of death (PLUD) confirmed that single KOs die at bacterial loads comparable to controls (~10^6^ CFU), whereas double and triple KOs exhibit high variability, with many individuals dying at significantly lower CFU values (10^4^–10^5^). This supports the conclusion that the cumulative loss of H_2_S-producing enzymes leads to death as a result of an immune-mediated pathology caused by excessive inflammatory tissue damage, rather than as a result of an uncontrolled infection. However, this interpretation should be regarded as preliminary in view of the lack of direct histological evidence.

### 4.2. Chronic Immune Activation Under Basal Conditions

H_2_S deficiency disrupts standard metabolic housekeeping, generating a state of chronic oxidative stress and hyperhomocysteinemia, which acts as a continuous, sterile danger signal. Our transcriptomic data revealed that this redox imbalance establishes a new, primed immune threshold under basal conditions. To counteract spontaneous autoinflammatory pathology, the organism upregulates a set of compensatory defense mechanisms. Thus, we observed significant basal induction of detoxification networks—such as Cytochrome P450 and Glutathione S-transferases (GSTs) (Figure S6)—and specific protective proteins, including serpins and Bomanins (Figure 4 and Figure 5).

Our transcriptomic analysis of KO lines demonstrates that H_2_S deficiency radically alters the transcriptional landscape of the entire innate immune system, stress responses, and several key metabolic pathways in *Drosophila*.

Under control conditions, double and triple KO flies exhibited increased expression of pattern recognition receptors (e.g., *PGRP-LC*, *PGRP-LA*), negative regulators (*PGRP-LF*, *PGRP-LB*), and stress-related genes (*Tak1*, *kayak*, *Mmp1*), but did not show an increase in most AMPs. This mismatch suggests that H_2_S deficiency drives the immune system of KO flies to a state of readiness. However, compensatory mechanisms (including increased amidase activity and serpin expression) prevent the development of spontaneous autoinflammatory pathology. Thus, the increased expression of serpin proteins such as Spn77Ba and Spn47C is likely to represent a protective mechanism against systemic inflammation and epithelial damage in these cell lines.

It is of note that Spn77Ba is the main inhibitor of melanization proteases, protecting tracheal and intestinal epithelia [48,58]; Spn47C, an ortholog of human SERPINH1/Hsp47 [88], may bind IRE1 under proteotoxic stress to prevent cell death.

The upregulation of cysteine-rich Bomanins (e.g., *BomBc1*, *BomS6*) and *Defensin* under control conditions is of particular interest. We hypothesize that these peptides function as a systemic “molecular shield”. By interacting with lipid rafts, they probably stabilize host cell membranes against the deleterious effects of reactive oxygen species (ROS) and lipid peroxidation [52,89], acting as a direct compensatory mechanism in KO lines for the loss of H_2_S-mediated antioxidant capacity.

### 4.3. Double and Triple Knockout: Immune Hyperactivation Caused by H_2_S Deficiency

The double knockout (*cbs*/*cse*) results in immune hyperactivation caused by H_2_S deficiency. These flies show the highest AMPs induction, a strong stress response (high *hsp70* levels, high *TotC* levels). This phenotype is consistent with a model in which H_2_S acts as an anti-inflammatory mediator [5] and its removal can lead to an uncontrolled immune response [11]. After septic injury, double KOs displayed a strong induction of IMD and Toll pathway components, including *Relish*, *Dif*, multiple serine proteases (*Hayan*, *Sp7*, *SPE*), and a broad array of AMPs (*Diptericins*, *Cecropins*, *Edin*, *Attacin C/B/D*). Notably, Attacin D—an atypical IMD effector that causes damage by aggregating within Malpighian tubule cells—was induced only in double and triple KOs, suggesting that renal pathology may contribute to the poor survival of these lines. This transcriptional hyper-reactivity was balanced by co-induction of negative regulators (*PGRP-SC2*, *Diedel*, *Necrotic*), indicating that feedback inhibition remains operational but may be insufficient to restore homeostasis.

The triple KO flies are of particular interest. Although in this line, the H_2_S level is similar to that of double KO flies [16], these flies exhibited poorer survival after bacterial infection and less pronounced AMPs induction in comparison with the double KO line. This paradox may be partially explained by our transcriptomic data. Thus, triple KO flies exhibit a significant upregulation of negative immune regulators, including *pirk*, *PGRP-LB*, and *PGRP-SC1/2*. This indicates that the immune system of this line has probably reached a state of exhaustion or active suppression. However, it appears that this suppression reduces the flies’ ability to mount an effective antimicrobial defense when necessary, leading to low survival rates in the event of septic infections. Besides, the KO of the *tst1* gene may exacerbate intoxication. The thing is that since TST1 is a sulfurtransferase involved in cyanide detoxification [90], we hypothesize that its removal may result in the accumulation of cyanide or other reactive forms of sulfur. In the context of an already disrupted transsulfuration pathway (for example, in the case of a double knockout), this causes additional metabolic stress, which is likely to impair the host’s viability and resilience, potentially exacerbating immune dysregulation. However, there is currently no direct biochemical evidence of cyanide accumulation in these flies, and this interpretation remains hypothetical until it is confirmed by direct measurements.

In this regard, this interpretation is supported by the peculiar characteristics of the *TST1-/-* KO flies. This line, deprived of the enzyme involved in detoxification processes, exhibits a surprisingly mild immune phenotype: in particular, its survival rate following septic injury was comparable to that of the control line, as was its basal transcriptome. However, septic injury induces multiple differences in the expression of immune-related genes in comparison with the control flies. This indicates that loss of *tst1*, while insufficient to cause chronic inflammation, does modulate the dynamics of the immune response upon infection, and its importance is unmasked under stress conditions.

It has also recently been shown that cyanide, produced endogenously in mammalian cells and tissues, performs various functions and provides cytoprotection at physiological concentrations [91]. Thus, its primary role in immunity may be indirect, by maintaining general metabolic health, but it becomes critically important when the transsulfuration pathway (*cbs*, *cse*) is already compromised.

### 4.4. Digestive Proteases: Transcriptomic Evidence for Leveling of Expression in Knockout Lines After Septic Injury

The insect gut serves not only as a digestive organ but also as a key component of the innate immune system [75,92], acting as the first line of defense against foodborne pathogens. Our data reveal that disruption of sulfur metabolism profoundly alters the transcriptional regulation of several digestive enzymes, with distinct genotype-specific patterns.

Under control conditions, double and triple knockout lines showed increased expression of trypsin-encoding genes and several Jonah family chymotrypsin-like serine proteases (*Jon66Cii*, *Jon44E*, *Jon74E*). However, in the triple KO, increased expression levels were observed for many other members of the Jonah family (including the Jon99, Jon25, and Jon65 subfamilies). This expanded *Jonah* response may reflect more severe gut dysbiosis or a broader compensatory metabolic adaptation in triple KOs, likely linked to *tst1* loss and cyanide toxicity. After septic injury, the control and *TST1-/-* KO lines exhibited a pronounced reduction in expression levels of both trypsin-encoding genes and Jonah proteases, reflecting the classical shift of energy expenditure from digestion to immunity [79]. A similar, though less pronounced, reduction was observed in the triple KO line. In contrast, no such decrease was observed in double KOs. It is important to note that when comparing the relative expression levels of these genes in infected double and triple KOs with those in the infected control line, their expression profiles continued to show elevated expression (Figure 6). This demonstrates that KO lines with low H_2_S maintain a stable homeostatic balance in digestive protease expression during the immune response.

Notably, double KO flies display elevated basal transcripts of *tobi*, *maltases*, and *ilp2*, suggesting that these flies experience chronic energy stress, likely due to oxidative damage and metabolic inefficiency. Following infection, all flies exhibit increased expression levels of *tobi* and *maltases*. The level of *ilp2* transcripts decreases in double and triple KO, but not in the control line, resulting in equally low expression levels across all lines. This observation suggests that the Toll-mediated suppression of insulin signaling in all lines redirects nutrients toward the immune defense.

### 4.5. Stress Pathway Activation

Basal elevation of *Tak1*, *kayak*, and *Mmp1* in double, triple KOs suggests that loss of transsulfuration metabolites primes stress-related signaling even without infection. The activation of JNK likely stimulates the expression of detoxification enzymes (e.g., CYP, GST, UGT) and TEP opsonins [65,67], contributing to the observed state of immune readiness. In double and triple KO lines, a more pronounced induction of *hsp70* is observed following a septic injury, indicating a greater degree of disruption in cellular proteostasis. Furthermore, the triple KO flies, which exhibited the lowest levels of *Turandot* induction *TotA*, *TotC*, *TotM*, *TotX*), also showed the greatest increase in *hsp70* levels following septic injury, which is consistent with reports that *Turandot* gene knockdown increases *hsp70* expression. This inverse correlation supports the model postulating that Turandot proteins act in the hemolymph to mitigate extracellular damage, analogous to the intracellular function of Hsps [86]. The failure of the triple KO to mount a proper Turandot response may contribute to its extreme sensitivity to infection.

## 5. Conclusions

H_2_S acts as a critical metabolic checkpoint that restrains the basal activation of immune, stress, and metabolic pathways. Its deficiency leads to the sustained activation of these pathways in non-infectious conditions, resulting in the development of chronic inflammation. This state of readiness leads to an overly active immune response during infection, while simultaneously triggering compensatory defense mechanisms (Bomanins, Serpins, Turandots) to limit collateral damage. These findings establish a new link between sulfur metabolism, immune homeostasis, proteostasis, and metabolic resilience in vivo. This work establishes that sulfur metabolism in *Drosophila* is not a monolithic regulator but a modular network whose disruption leads to distinct immunopathological states. H_2_S produced by CBS/CSE is a key anti-inflammatory signal. Importantly, the observed immune dysregulation is primarily driven by H_2_S deficiency rather than by homocysteine accumulation. This conclusion is supported by the fact that the single KO *CBS*^−^/^−^ line, despite exhibiting hyperhomocysteinemia comparable to the double KO, retains higher H_2_S levels and shows a significantly milder phenotype after infection. The TST1 enzyme plays a key role in metabolic detoxification, the importance of which becomes evident when the transsulfuration pathway is disrupted. These findings provide a conceptual framework for a deeper understanding of human conditions such as severe hyperhomocysteinemia or combined metabolic disorders, which may be accompanied by immune dysfunction and systemic toxicity. The accumulated results argue for therapeutic strategies that not only modulate H_2_S levels but also support broader sulfur metabolic flux and detoxification pathways to restore holistic immune-metabolic homeostasis.

## 6. Limitations

1. Transcriptional level only, one 6 h time point in transcriptomic library: Our conclusions are primarily based on transcriptomic data. Protein abundance, enzymatic activity, and functional outcomes were not directly measured.
2. Single pathogen: Responses to other pathogens may differ.
3. The absence of transcriptomic data for *CBS-/-* and *CSE*-/- KO lines limits our ability to fully dissect the contributions of H_2_S deficit versus homocysteine at the transcriptional level.
4. Suggested TST1 toxicity mechanism is speculative: Suggested cyanide accumulation in triple KO flies requires direct biochemical validation. Thus, direct measurements of endogenous cyanide levels or pharmacological rescue experiments are needed to test this hypothesis.
5. To prove the statement that combined KO flies die from immune-mediated pathology rather than uncontrolled bacterial proliferation, direct evidence (e.g., histopathology, inflammatory markers) is required.
6. Gut microbiota not profiled: Changes in Jonah proteases suggest dysbiosis, but this was not directly tested.

Despite these limitations, our data for the first time provide a comprehensive transcriptional map of how H_2_S deficiency remodels innate immunity, stress responses, carbohydrate metabolism, and insulin-like peptide expression in *Drosophila*. This study provides a framework for understanding human conditions when sulfur metabolism is disrupted, such as hyperhomocysteinemia or combined metabolic disorders, when immune dysfunction and systemic toxicity coexist.

## Data Availability

The original data presented in the study are openly available in [NCBI GEO database] at [https://www.ncbi.nlm.nih.gov/geo/query/acc.cgi?acc=GSE333980 [GSE333980]] (accessed on 7 July 2026).

## Supplementary Materials

The following supporting information can be downloaded at https://www.mdpi.com/article/10.3390/antiox15070881/s1: Table S1: List of primers used for qRT-PCR experiments; Figure S1: Growth kinetics of *Bacillus subtilis 168* in wild-type (58492), single-deletion mutants (*cbs*-/-, *cse*-/-, *tst1*-/-), and multiple-deletion mutants (double/triple KO); Figure S2: The scatterplot showing the LogFC values of the key genes examined in this study demonstrating that removing of the outlier sample do not affect differential expression. Comparison of *B. subtilis* (B.S) infected double KO flies to intact 58492 flies. X-axis–double KO include 3 samples, Y-axis–double KO include 2 samples; Figure S3: Box plots illustrating differences in the expression of genes involved in the immune response; Figure S4: Box plots showing differences in Serpin expression under control conditions and following septic injury; Figure S5: Box plots showing differences in the expression of opsonins and lectins under control conditions and following septic injury; Figure S6: Heat map of detoxification system components with varying expression levels in intact KO flies and after septic injury with *B. subtilis*.
