# Supplementary material for: Knockouts of Sulfur Metabolism Genes Induce Chronic Inflammation and Immune Dysregulation in Drosophila melanogaster
Source: Antioxidants (Basel). 2026 Jul 16;15(7):881. doi: 10.3390/antiox15070881 (PMC13405187; doi:10.3390/antiox15070881)
Supplement: Supplementary file 1 [file antioxidants-15-00881-s001.zip › antioxidants-4402409-supplementary.pdf]

Supplementary material

**Primer name**	**Sequence**
Relish Fwd	5’-ACTCACCATACCAGAATCAGC-3’
Relish Rev	5’-TCGCCGTCAGATGCCAGG-3’
Drosocin Fwd	5’-GTTCACCATCGTTTTCCTGCT-3’
Drosocin Rev	5’-ACACATCTTTAGGCGGGCAG-3’
Diptericin B Fwd	5’-AAGCCCAAAGCAAGGATTCGAT-3’
Diptericin B Rev	5’-GCTGTTGCCATAGGGTCCAC-3’
Attacin A Fwd	5’-GACCAAAACACACACGCCCG -3’
Attacin A Rev	5’-CTGGGAAGTTGCTGTGCGTC-3’
Dif Fwd	5’-TTGAAACGCCGCCAGGAACT-3’
Dif Rev	5’-CATTTTCTTGACGGCTATCTC-3’
Drosomycin Fwd	5’-TACTTGTTCGCCCTCTTCGC-3’
Drosomycin Rev	5’-TTCGCACCAGCACTTCAGAC-3’
Bomanin Bc1 Fwd	5’-GTGCCTGATTCTGTCCTTTGC-3’
Bomanin Bc1 Rev	5’-CCTCCTCCGCCGCCAGC-3’
Defensin Fwd	5’-CTTCGTTCTCGTGGCTATCG-3’
Defensin Rev	5’-GCTTCTGGCGGCTATGCTG-3’
Unpaired 3 Fwd	5’-AGAAATTGAATGCCAGCAGTAC-3’
Unpaired 3 Rev	5’-GGCCAGCTTGTCCGCATTG-3’
Tourandot A Fwd	5’-CCAAAATCAATAGCACCCAGGAA-3’
Tourandot A Rev	5’-CCTTCACACCTGGAGATACAAT-3’
Tourandot C Fwd	5’-CCTGCTCCTGATTAGTCCTTT-3’
Tourandot C Rev	5’-GCTGCCACCGAGTTCTTCAA-3’
Hsp70 Fwd	5’-CTCAGAACAGCAGCTGAACG-3’
Hsp70 Rev	5’-GATGTCGTGGATCTGACCCT-3’
RpL32 Fwd	5’-CCATCCGCCCAGCATACAG-3’
RpL32 Rev	5’-CGGTGGGCAGCATGTGGC-3’
Bacill.Subt. Fwd	5′-CCAGTAGCCAAGAATGGCCAGC-3′
Bacill.Subt. Rev	5′-GGAATAATCGCCGCTTTGTGC-3′

Table S1. List of primers used for qRT-PCR experiments.

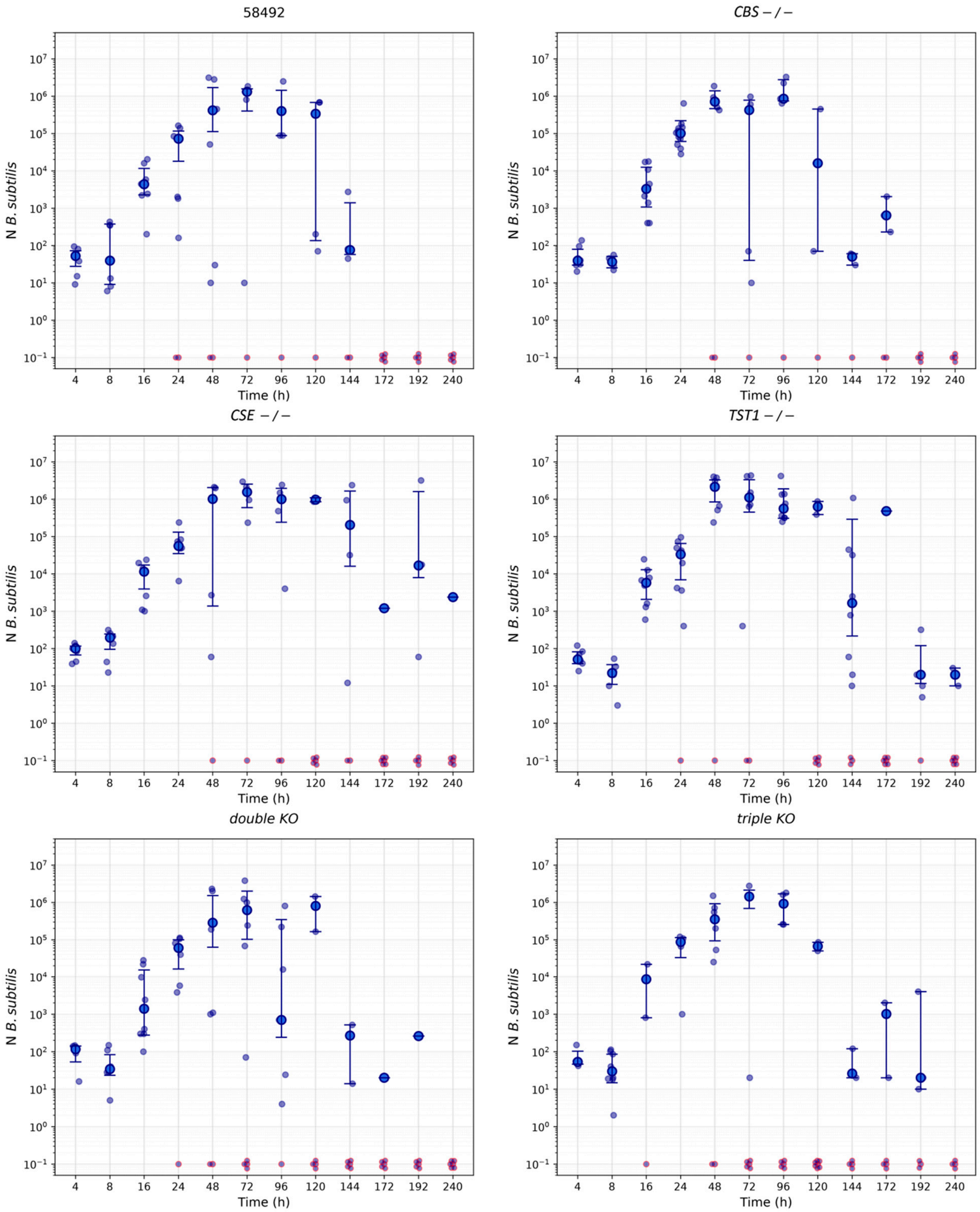

Figure S1. Growth kinetics of *Bacillus subtilis 168* in wild-type (58492), single-deletion mutants (*cbs* -/-, *cse* -/-, *tst1* -/-), and multiple-deletion mutants (double/triple KO). Each data point reflects a pooled sample of three flies. Error bars reflect the median and the interquartile range (IQR) from the median. Bacterial load (N *B. subtilis*) is displayed on a logarithmic scale across 240 hours post-infection. Time points with no detectable growth are plotted at the lower limit of detection (10^{-1}). Each genotype is presented in a separate panel, representing independent biological replicates (n ≥ 4 per time point).

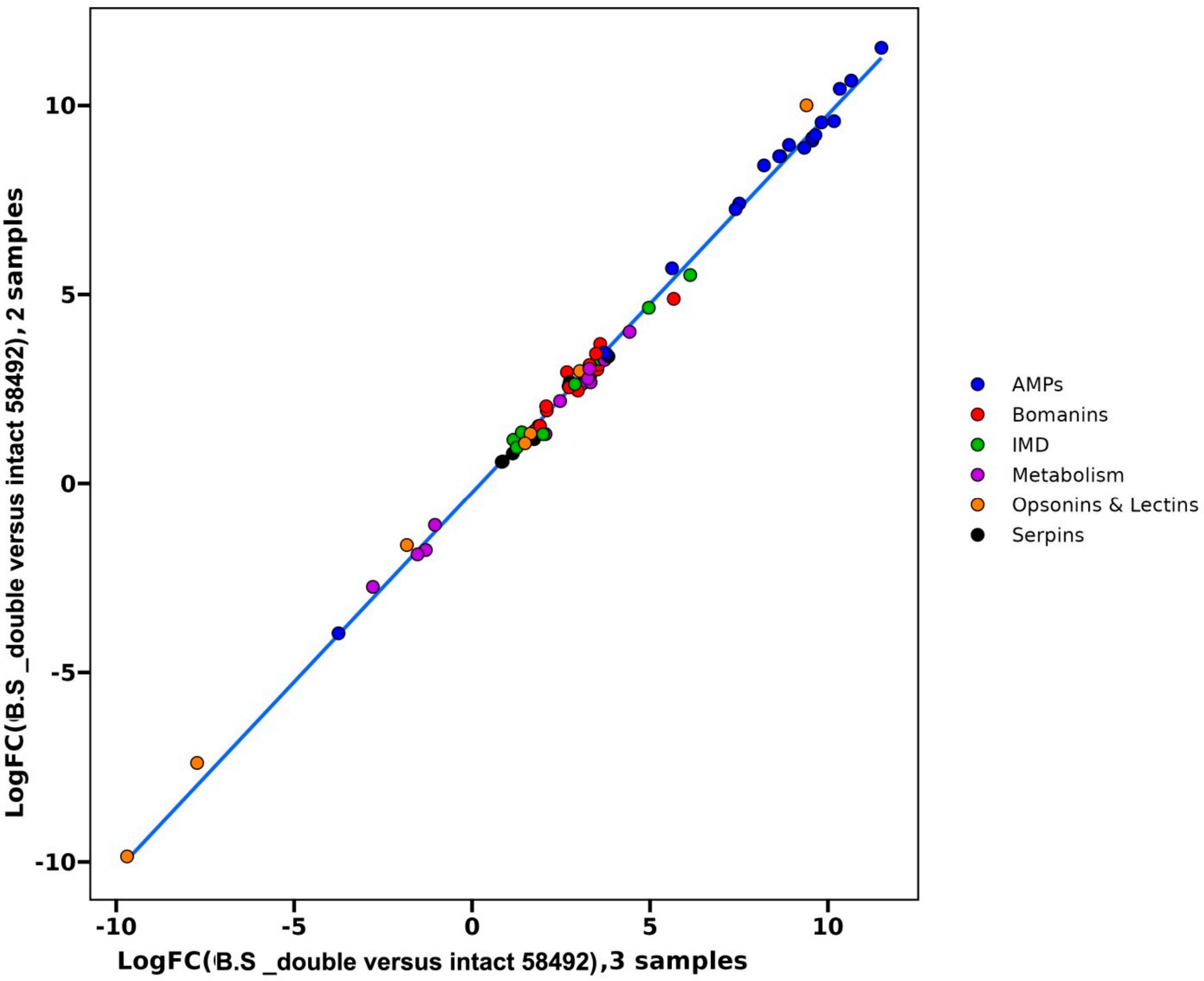

Figure S2. The scatterplot showing the LogFC values of the key genes examined in this study demonstrating that removing of the outlier sample do not affect differential expression. Comparison of *B. subtilis* (B.S) infected double KO flies to intact 58492 flies. X-axis – double KO include 3 samples, Y-axis – double KO include 2 samples.

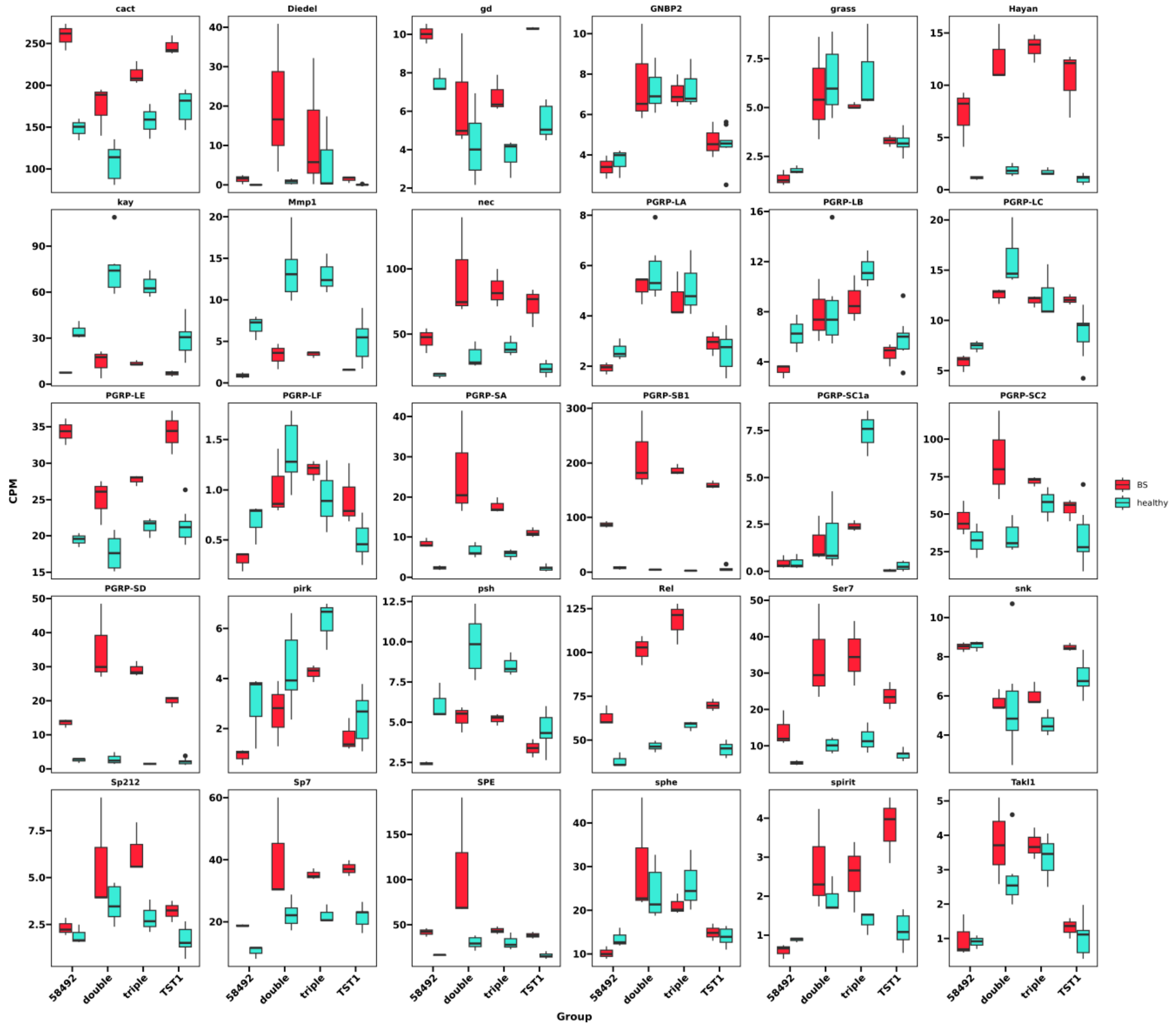

Figure S3. Box plots illustrating differences in the expression of genes involved in the immune response. The x-axis represents the control line (58492), a single *tst1* gene deletion (TST1), and multiple gene knockouts (double and triple). Color coding corresponds to injected *B. subtilis* (red rectangles) and uninfected flies (blue rectangles). In the box plots, the center line represents the median, the rectangle represents the interquartile range (IQR), and the whiskers represent the minimum and maximum values. Expression levels are presented as normalized values per million. All genes in the box plots have significant differential expression (FDR ≤ 0.05) in at least one of the comparisons used in Figure 4

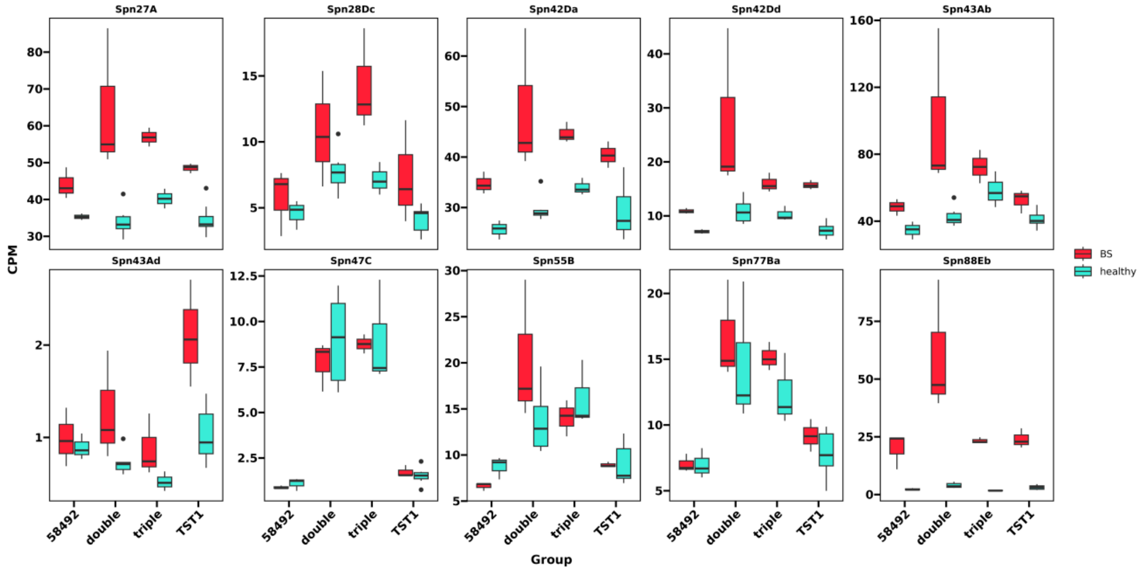

Figure S4. Box plots illustrating differences in the expression of Serpin genes under control conditions and following septic injury. The x-axis represents the control line (58492), a single *tst1* gene deletion (TST1), and multiple gene knockouts (double and triple). Color coding corresponds to injected *B. subtilis* (red rectangles) and uninfected flies (blue rectangles). In the box plots, the center line represents the median, the rectangle represents the interquartile range (IQR), and the whiskers represent the minimum and maximum values. Expression levels are presented as normalized values per million. All genes in the box plots have significant differential expression (FDR ≤ 0.05) in at least one of the comparisons used in Figure 5.

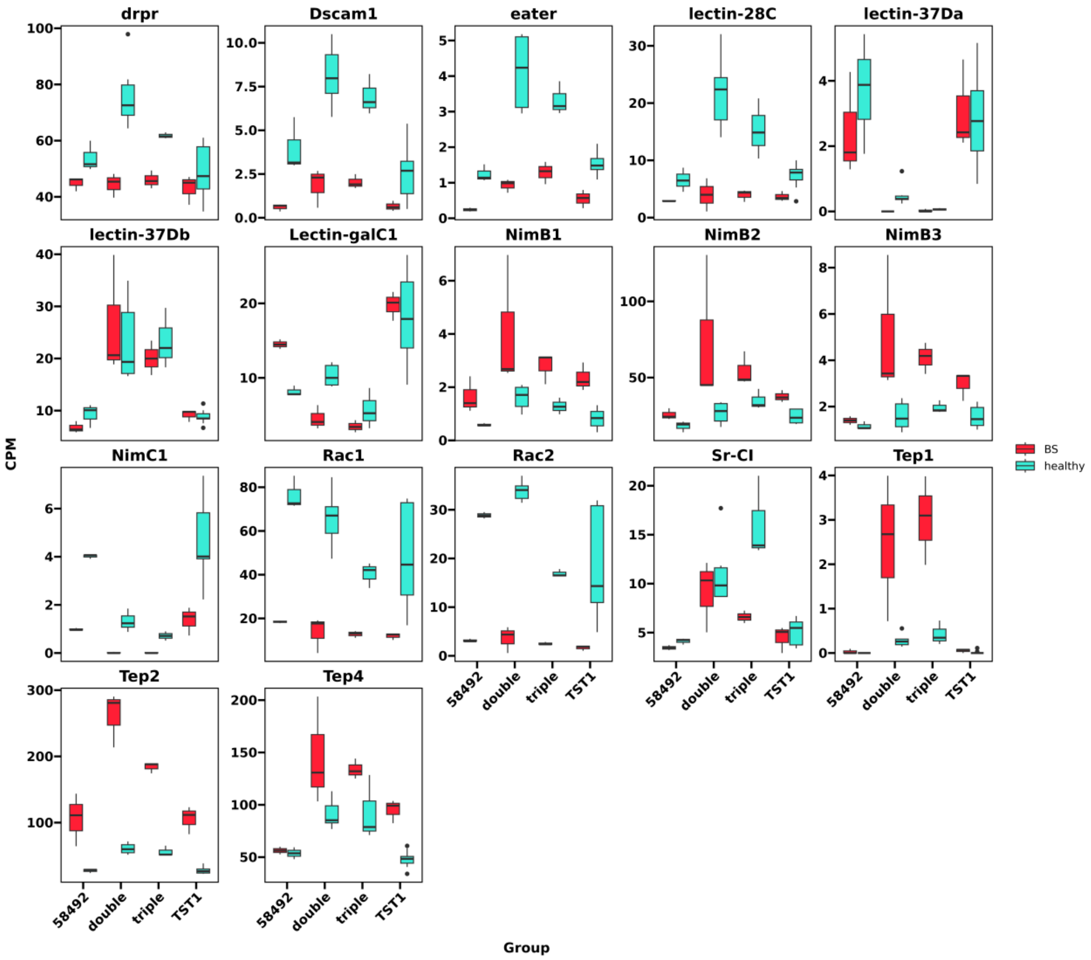

Figure S5. Box plots showing differences in the expression of opsonins and lectins under control conditions and following septic injury. The x-axis represents the control line (58492), a single *tst1* gene deletion (TST1), and multiple gene knockouts (double and triple). Color coding corresponds to injected *B. subtilis* (red rectangles) and uninfected flies (blue rectangles). In the box plots, the center line represents the median, the rectangle represents the interquartile range (IQR), and the whiskers represent the minimum and maximum values. Expression levels are presented as normalized values per million. All genes in the box plots have significant differential expression (FDR ≤ 0.05) in at least one of the comparisons used in Figure 5.

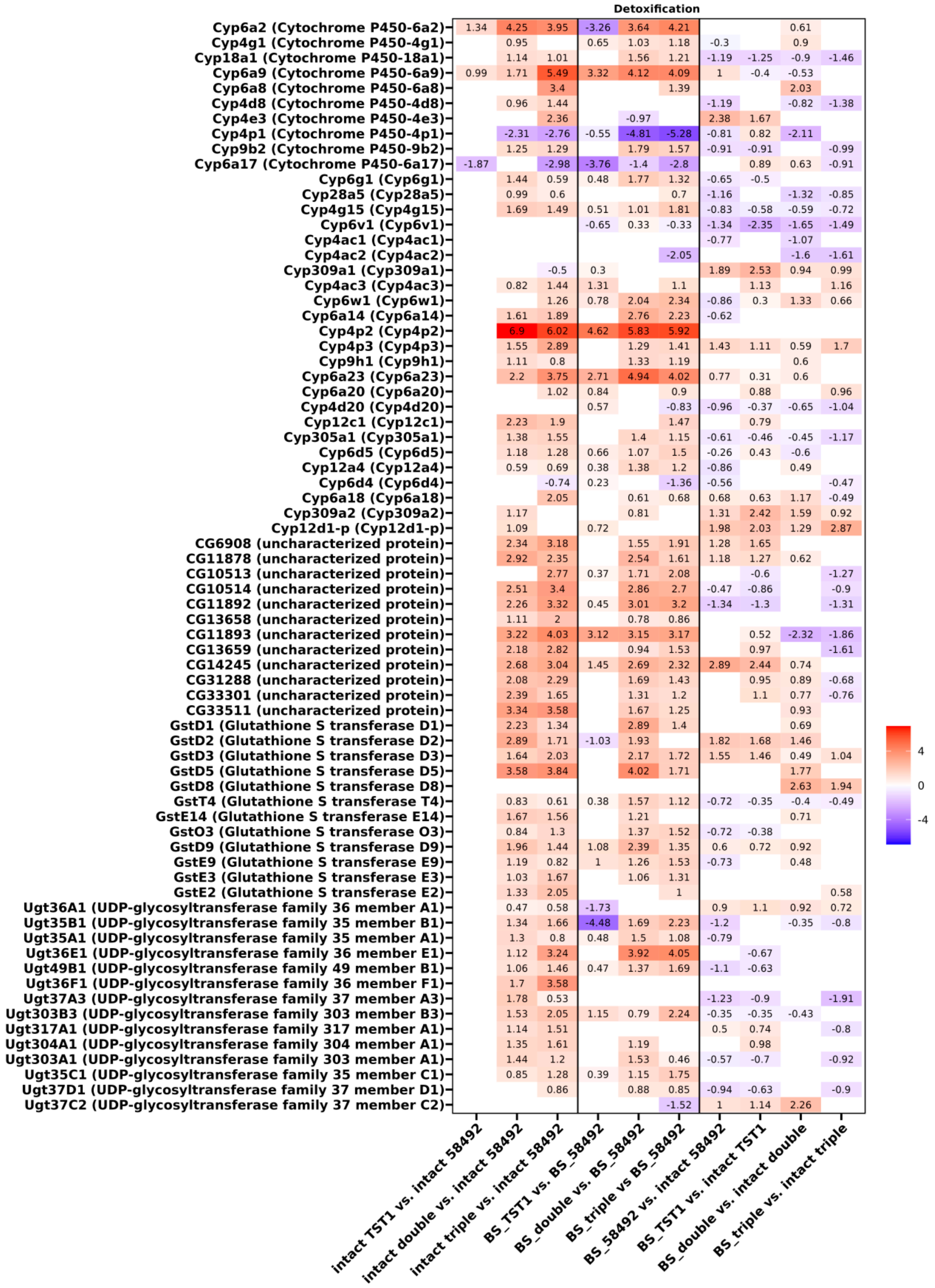

Figure S6. Heat map of detoxification system components with varying expression levels in intact KO flies and after septic injury with *B. subtilis*.

Three pairwise comparisons were done: 1. Intact (healthy) KO flies to control 58492 intact flies. 2. Infected *B. subtilis* (BS) KO flies to infected *B. subtilis* 58492 flies. 3. *B. subtilis* infected flies to intact flies of the same genotype. The heatmaps display the logFC values for the genes of interest: blue indicates genes with reduced expression (logFC < 0), whilst red indicates genes with increased expression (logFC > 0).
